# Temporal patterns of melanopsin-evoked calcium signaling engage NF-κB-associated transcriptional and phosphoproteomic responses

**DOI:** 10.1016/j.isci.2026.118031

**Published:** 2026-10-26

**Authors:** Parvaneh Nikpour, Manuel Varas-Godoy, Johan Tenghamn, Per Uhlén, Erik Smedler

**Affiliations:** 1 Department of Psychiatry and Neurochemistry, Institute of Neuroscience and Physiology, Sahlgrenska Academy at University of Gothenburg, Gothenburg, Sweden; 2 Department of Genetics and Molecular Biology, Faculty of Medicine, Isfahan University of Medical Sciences, Isfahan, Iran; 3 Centro de Biología Celular y Biomedicina (CEBICEM), Facultad de Ciencias, Universidad San Sebastián, Santiago, Chile; 4 Centro Ciencia & Vida, Fundación Ciencia & Vida, Santiago, Chile; 5 Department of Medical Biochemistry and Biophysics, Karolinska Institutet, Stockholm, Sweden; 6 The Wallenberg Centre for Molecular and Translational Medicine, Gothenburg, Sweden; 7 Department of Clinical Chemistry, Sahlgrenska University Hospital, Gothenburg, Sweden; 8 Department of Psychiatry for Affective Disorders, Sahlgrenska University Hospital, Gothenburg, Sweden

**Keywords:** calcium signaling, frequency decoding, transcriptomics, phosphoproteomics, MYC

## Abstract

Cells use oscillatory calcium (Ca^2+^) signals to regulate diverse biological processes, with temporal dynamics providing a potential layer of intracellular information. We used optogenetic activation of the Gαq-coupled photoreceptor melanopsin to impose defined slow or fast temporal stimulation patterns in human non-excitable cells, evoking oscillatory Ca^2+^-associated signaling while preserving receptor-proximal signaling. Fast, regular melanopsin stimulation induced stronger nuclear factor κB (NF-κB)-associated tumor necrosis factor (*TNF*) and interleukin-8 (*IL8*) expression than temporally irregular stimulation, while *TNF* and *IL8* showed partly distinct responses to stimulation frequency. Transcriptomic analysis identified an MYC-associated gene expression program despite unchanged *MYC* transcript levels. Integration of transcriptomic and phosphoproteomic datasets identified candidate kinase networks involving PRKDC, CHEK2, and ATM, potentially linking temporally patterned signaling to cell-cycle and stress-response pathways. Together, these findings show that distinct temporal patterns of melanopsin-evoked signaling are associated with distinct transcriptional and phospho-signaling responses, highlighting temporal dynamics as a feature of intracellular information processing.

## Highlights

• Temporal patterns of melanopsin stimulation elicit distinct transcriptional responses
• *TNF* and *IL8* show distinct sensitivities to stimulation frequency and temporal regularity
• Transcriptomics identifies an MYC-associated gene expression signature
• Phosphoproteomics identifies candidate kinase networks associated with temporal signaling

## Graphical abstract

**Figure:**
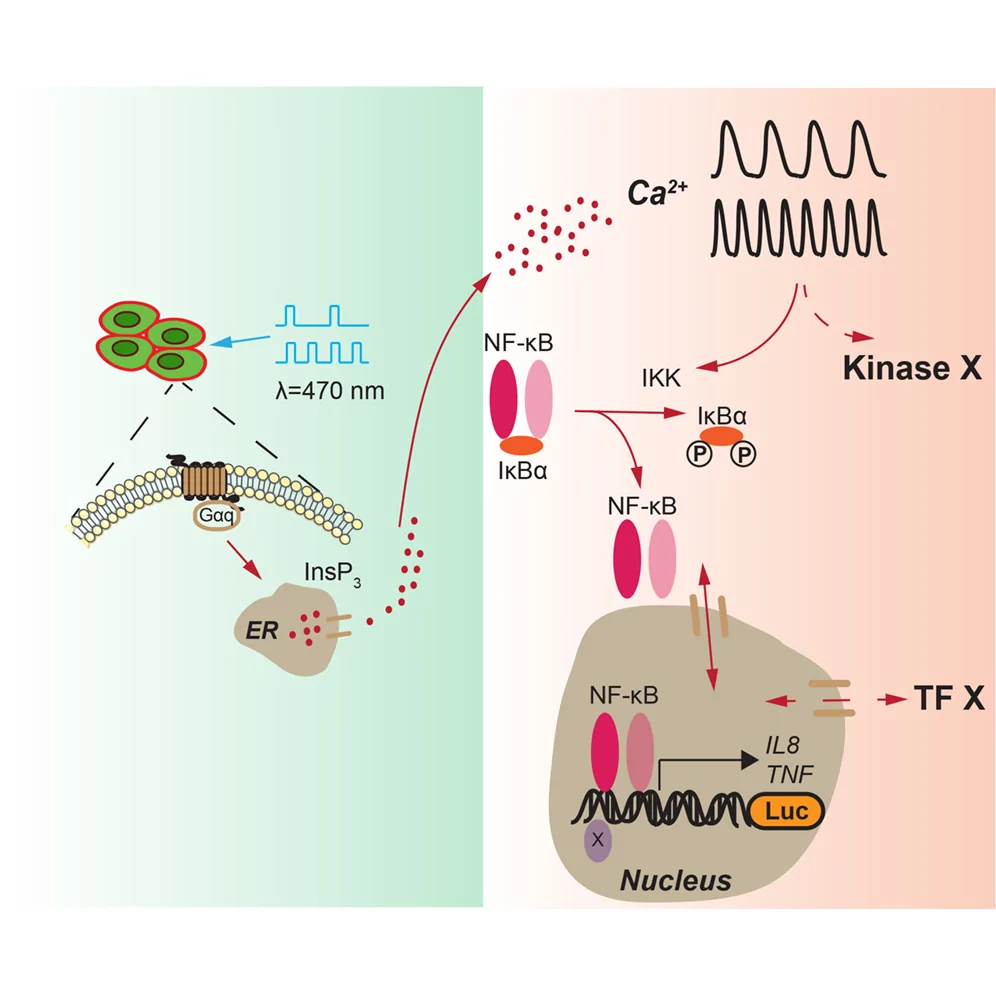

## Teaser

Biological sciences; Cell biology; Transcriptomics

## Introduction

Signaling occurs in all cells to regulate intracellular processes and to communicate their state to neighboring cells. Each cell has around 20 distinct intracellular signal transduction pathways with a multitude of interactions.^1^ Among these, several are related to Ca^2+^ signaling (for instance, cyclic ADP-ribose signaling, voltage-/receptor-operated channels, and pathways involving activation of phospholipase C), which is involved in a wide array of processes, including fertilization, apoptosis, proliferation, differentiation, muscle contraction, and learning.^1^^,^^2^^,^^3^ The remarkable versatility of Ca^2+^ as an information mediator stems from its extensive spatial and temporal diversity, allowing it to generate highly varied patterns.^4^ Temporarily, Ca^2+^ concentrations can oscillate periodically, driven by the activity of numerous transporters.^3^ Modulation of Ca^2+^ oscillatory frequency has been shown to regulate various cellular functions. For example, in the developing brain, ambient gamma-aminobutyric acid (GABA) and glutamate stimulate interneuron migration. Upon reaching the cortex, upregulation of the potassium-chloride cotransporter KCC2 reduces the frequency of spontaneous Ca^2+^ activity, thereby reducing migration.^5^ In post-migratory pyramidal neurons, the frequency of Ca^2+^ oscillations increases as the number of branching dendrites increases.^6^ Gain-of-function mutations in SHP-2/PTPN11, associated with the developmental disorder Noonan syndrome, enhance the frequency-dependent control of NFAT (nuclear factor of activated T cells) by Ca^2+^ oscillations, potentially contributing to congenital heart defects.^7^ In pancreatic β cells, the frequency of Ca^2+^ oscillations governs mitochondrial ATP synthesis, which in turn drives insulin secretion.^8^ Similarly, glioma cells form functional networks and exhibit spontaneous Ca^2+^ oscillations that are fine-tuned to promote proliferation, potentially via NF-κB (nuclear factor kappa-light-chain-enhancer of activated B cells).^9^ This activity is similar to the dynamics of developing neural progenitors derived from mouse embryonic stem cells.^10^^,^^11^

Biological information encoded in Ca^2+^ oscillations is decoded by various intracellular molecules that adjust their activity in response to these signals.^12^ Several molecular mechanisms have been proposed for frequency decoding, with a handful of specific decoders identified to date. These include NF-κB,^13^ nuclear factor of activated T cells (NFAT),^13^^,^^14^ MAPK (mitogen-activated protein kinase),^15^ PKC (protein kinase C),^16^ CaMKII (Ca^2+^/calmodulin-dependent protein kinase II),^17^ and calpain.^18^ NF-κB and NFAT predominantly decode low-frequency signals in the range of 1–100 mHz, while CaMKII operates in the higher frequency range of 0.1–1 Hz.^18^ This separation in the frequency domain is mainly explained by NF-κB and NFAT being shuttled to and from the nucleus as the underlying mechanism of frequency decoding,^19^ while decoding by CaMKII is dependent on autophosphorylation regulated by Ca^2+^ and calmodulin binding.^17^

Optogenetics refers to the use of light to control genetically modified cells that express light-sensitive proteins.^20^^,^^21^ Typically, excitable cells such as neurons are activated or inhibited by light stimulation of different versions of the cation channel channelrhodopsin-2 or the chloride pump halorhodopsin. Melanopsin, a G_αq_-protein-coupled receptor encoded by the *OPN4* gene in humans, is expressed in intrinsically photosensitive retinal ganglion cells and plays a critical role in regulating circadian rhythms.^22^ Light in the blue spectrum is absorbed by the vitamin A derivate retinal bound to melanopsin, leading to its isomerization from 11-cis-retinal to all-trans-retinal.^23^ Upon activation, melanopsin triggers the release of Ca^2+^ from the endoplasmic reticulum (ER) through the inositol 1,4,5-trisphosphate (InsP_3_) receptor (InsP_3_R).^24^ Previously, melanopsin has been expressed in non-excitable cells to enable light-controlled regulation of NFAT activity.^25^

In this study, we assay transcriptomic and phosphoproteomic responses to melanopsin stimulation patterns in the millihertz range, in contrast to previous studies that focused on specific decoders to assay their dynamics. We utilized heterologous expression of melanopsin to impose defined temporal patterns of stimulation that evoke repetitive Ca^2+^ responses and to investigate downstream temporal decoding in a non-excitable cell line. We analyzed NF-κB-associated transcriptional responses and found that tumor necrosis factor (*TNF*) and interleukin-8 (*IL8*) showed distinct sensitivities to the temporal stimulation pattern. We predicted a network of frequency-dependent genes associated with MYC through bulk RNA sequencing. Finally, phosphoproteomics identified differential phosphorylation patterns and candidate kinase networks potentially linking temporally patterned melanopsin-evoked signaling to cell-cycle and stress-response pathways.

## Results

### Optogenetic induction of repetitive Ca^2+^ responses

To establish an optogenetic system for generating repetitive Ca^2+^ signaling, we developed a computer-controlled light-stimulation setup based on melanopsin activation (Figure 1A). The Gα_q_-protein-coupled receptor melanopsin, activated by blue light and co-expressed with mCherry, was introduced into non-excitable HeLa cells. Cells were transduced with lentiviruses with a construct carrying the melanopsin gene *OPN4* co-expressed with mCherry using an IRES sequence (Figure 1B). Simultaneous detection of the intracellular Ca^2+^ level was performed using the ratiometric dye Fura-2/AM. To activate melanopsin, we used a blue LED that emitted light of 470 nm. For live imaging of the kinetics and the influence of the melanopsin receptor on cellular Ca^2+^ levels, we used a setup with one single LED (Figure S1A), whereas six LEDs connected in series (Figure S1B) were used for experiments applying defined temporal melanopsin stimulation regimes inside the incubator. Various driving voltages were applied to control the radiant power of the LEDs. Measuring the light emitted from one single LED versus one LED connected with series of six revealed similar radiant powers applying 4.3 V and 17 V, respectively (Figures S1C and S1D). Testing the response of cells that overexpress melanopsin resulted in a prominent Ca^2+^ increase following 5 s of stimulation with the blue LED (Figure 1C). The Ca^2+^ response was consistent with involvement of phospholipase C (PLC) and InsP_3_R, since 5 μM of U73122 (Figure S2A) and 100 μM of 2-APB (Figure S2B) could inhibit the response. Cells transduced with viruses carrying only the empty vector were not sensitive to the blue LED light (Figure 1D).

**Figure 1:**
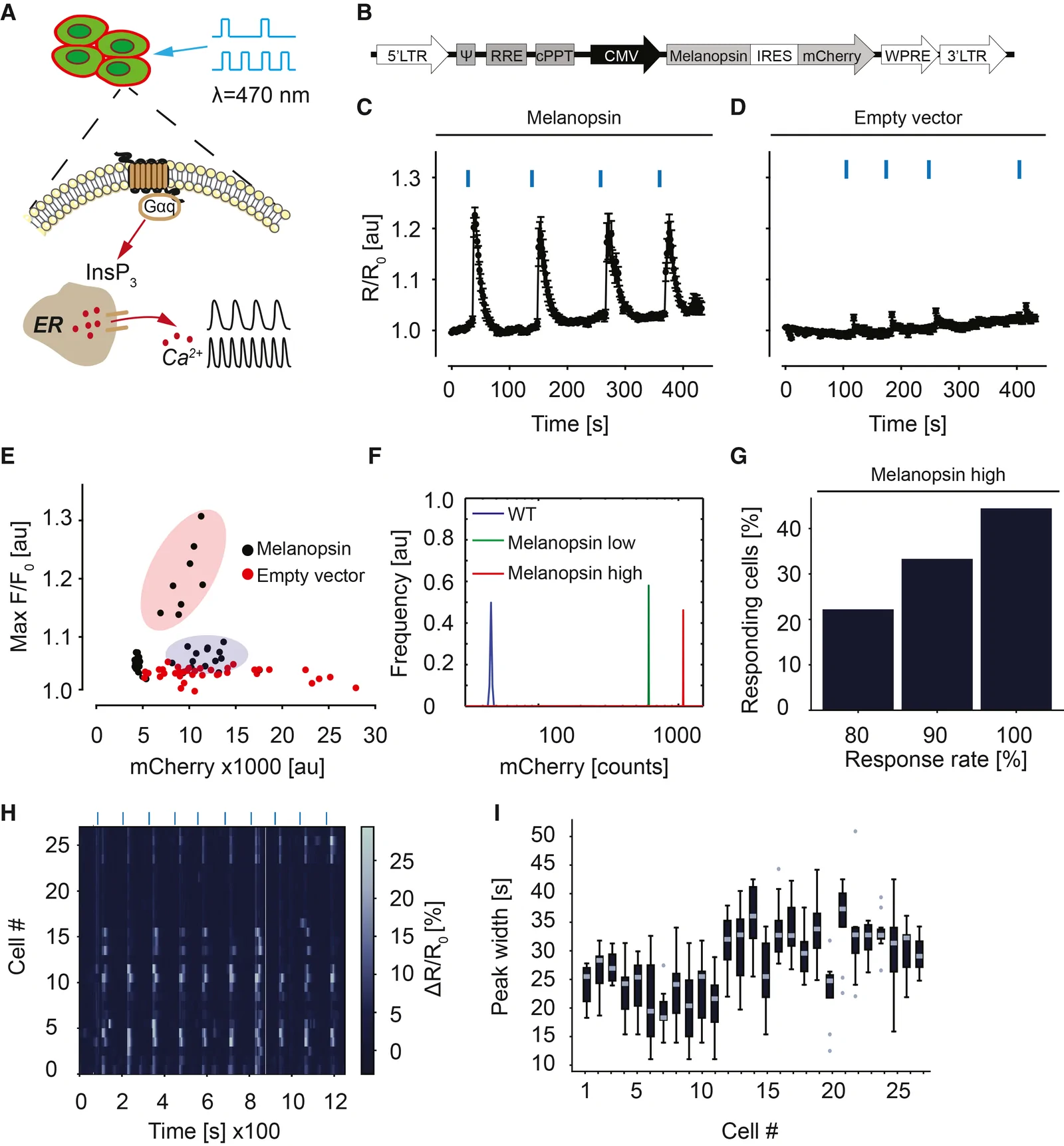
Optogenetic control of intracellular calcium signaling (A) Schematic of the experimental setup using optogenetics to control Ca^2+^ signaling. (B) The lentiviral expression vector for melanopsin. (C) Ca^2+^ trace for the average and SEM for five cells transduced with melanopsin expression vector. Blue markings denote 5 s of light stimulation. (D) Same as in (C) for cells transduced with empty vector. (E) Scatterplot of peak Ca^2+^ response to light stimulation as a function of mCherry intensity. Each dot represents one cell. Red region represents cells with Ca^2+^ response increasing with mCherry fluorescence, whereas blue region represents non-responders. (F) Distribution of mCherry intensity for the three sorted cell populations. (G) Number of cells responding with certain response rate for repeated light-stimulations. (H) Long-term Ca^2+^ imaging for repeated light stimulations. (I) Widths of the Ca^2+^ peaks evoked by light stimulation. Each boxplot represents the peak widths from a single cell, showing the median, the 1st, and 3rd quartiles (box edges) and whiskers extending to the furthest point within 1.5× the interquartile range. See also Figures S1 and S2

Both 5 and 4.3 V of driving voltage for the LED led to a clear Ca^2+^ response (Figure S2C). In a population of unsorted cells stimulated with blue light, two different subgroups appeared (Figure 1E). In one subgroup (blue region), cells lacked light sensitivity independent on mCherry fluorescence. In the other subgroup (red region), cells with stronger mCherry fluorescence responded with stronger Ca^2+^ influx than cells with weaker mCherry. These data show that we indeed could control the intracellular Ca^2+^ concentration with optogenetics.

### Stable cell lines expressing melanopsin

Next, we sought to establish stable melanopsin-expressing cells. Since there were differences in the Ca^2+^ response depending on the mCherry expression (Figure 1E), transduced cells were sorted by flow cytometry into three groups: empty vector, low melanopsin expression, and high melanopsin expression (Figure 1F). The mCherry fluorescence corresponded to the melanopsin expression, as validated by RT-qPCR with *OPN4* primers (Figures S2D and S2E). To determine if long-term light stimulation was harmful to the cells, we exposed our cells to light pulses for 5 s every minute for 12 h. This stimulation was not toxic to cells, as the morphology was unaffected (Figure S2F) and no increase in the early apoptotic marker Annexin V was observed (Figures S2G and S2H). Similar results were observed for cells expressing empty vector, low melanopsin, or high melanopsin. To control for potential low-level light toxicity, all subsequent experiments were normalized to empty-vector and unstimulated control cells, thereby accounting for both transgene expression and light exposure. Thus, neither the light itself nor the resulting Ca^2+^ influx caused apoptosis. In the melanopsin-high-expressing cell line, around 40% of the cells had a 100% response rate (around 30% had 90% response rate and around 20% had 80% response rate) upon repeated light stimulation for 20 minutes (Figures 1G and 1H). Light-evoked Ca^2+^ responses were clearly synchronous between cells (Figure 1H), although the peak width of the resulting Ca^2+^ signal upon repeated light stimulation slightly varied for each cell but was in general around 20–40 s (Figure 1I).

### NF-κB-associated responses to different melanopsin stimulation frequencies

To validate our experimental system, we first focused on NF-κB, which is known to decode the frequency of Ca^2+^ oscillations.^13^ To examine how NF-κB-associated transcription responds to different melanopsin stimulation frequencies, we used a luciferase-based reporter assay. Cells were stimulated with light either once every minute (15 mHz) or once every second minute (8 mHz) for 1 h. To test the need for co-factors, culture medium was supplemented in half of the samples with 100 nM of the melanopsin co-factor all-*trans* retinal (ATR). Firefly luciferase activity was normalized to Renilla luciferase and expressed relative to unstimulated cells with empty vector and without ATR (Figure 2A). Factorial ANOVA revealed significant effects of stimulation condition (F(2,40) = 27.71, *p* = 2.8 × 10^−8^), melanopsin expression level (F(2,40) = 40.00, *p* = 2.9 × 10^−10^), and ATR supplementation (F(1,40) = 8.97, *p* = 0.0047) on NF-κB-associated transcriptional activity. Significant interaction effects were also observed between stimulation and melanopsin expression (F(4,40) = 11.52, *p* = 2.6 × 10^−6^), stimulation and ATR supplementation (F(2,40) = 16.72, *p* = 5.3 × 10^−6^), and melanopsin expression and ATR supplementation (F(2,40) = 8.96, *p* = 6.1 × 10^−4^). Baseline differences between melanopsin expression groups and ATR conditions suggest that melanopsin expression and/or retinal supplementation may modestly influence basal NF-κB reporter activity. Therefore, interpretation focuses primarily on within-condition stimulation effects. NF-κB-associated transcriptional activity increased with melanopsin stimulation frequency, with the faster stimulation regime producing stronger reporter activity (Figure 2B). The light-induced increase in NF-κB-associated reporter activity was within a similar order of magnitude as acute lipopolysaccharide (LPS-induced activation of the reporter system (Figure S3A).

**Figure 2:**
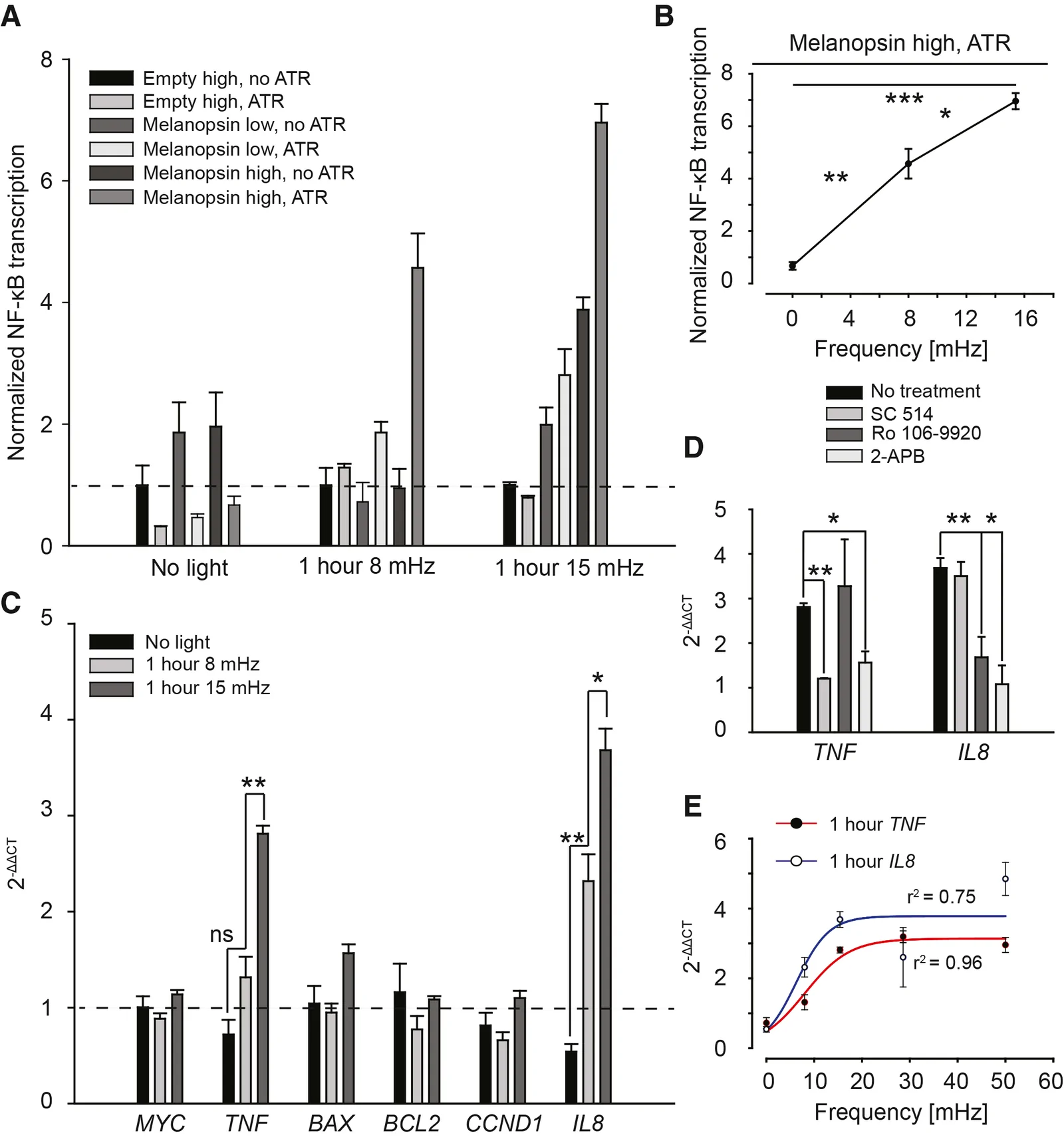
NF-κB-associated responses to different melanopsin stimulation frequencies (A) NF-κB-associated transcription as measured by luciferase assay. Firefly luciferase activity was normalized to Renilla luciferase and expressed relative to unstimulated controls. (B) Values from (A) only for experiments using the Melanopsin high cells with added ATR as a function of frequency. (C) Gene expression of a set of genes dependent on NF-κB as measured by RT-qPCR. (D) Effects of the IP3R inhibitor 2-APB (100 μM) and the NF-κB pathway inhibitors SC-514 (50 μM) and Ro 106–9920 (10 μM) on *TNF* and *IL8* induction. SC-514 preferentially reduced *TNF* induction, whereas Ro 106-9920 more clearly reduced *IL8* induction; 2-APB reduced induction of both genes. (E) Expression of *TNF* and *IL8* as function of frequency for a wider span. *p* values were calculated using factorial ANOVA followed by Tukey’s post hoc multiple-comparison test; average and SEM for *n* = 3 for independent cultures. ∗ *p* <0.05, ∗∗ *p* <0.01, and ∗∗∗ *p* <0.001. Stars in (B) indicate selected post hoc comparisons within melanopsin-high cells supplemented with ATR. See also Figure S3.

Next, a set of genes known to be dependent on NF-κB was assayed (*MYC*,^26^ *TNF*,^27^*BAX*,^28^ *BCL2*,^29^ *CCND1*,^30^ and *IL8*^31^) using RT-qPCR. Only the melanopsin high cell line with added ATR was used. *TNF* expression was significantly increased following the fast but not the slow stimulation regime (*p* = 0.00607 and *p* = 0.13868, respectively, *N* = 3), whereas *IL8* expression was significantly increased following both fast and slow stimulation (*p* = 0.00757 and *p* = 0.03606, respectively, *N* = 3) (Figure 2C). To assess the contribution of NF-κB-associated signaling to gene induction, cells were incubated with either 50 μM SC-514 (IKKβ inhibitor) or 10 μM Ro 106-9920 (IκBα ubiquitination inhibitor). SC-514 preferentially reduced *TNF* induction (*p* = 0.003475, *N* = 3), whereas Ro 106-9920 more clearly reduced *IL8* induction (*p* = 0.00211, *N* = 3) (Figure 2D), indicating partially divergent or gene-specific regulatory effects. In addition, treatment with 100 μM of the InsP3R inhibitor 2-APB reduced induction of both *TNF* and *IL8* (*p* = 0.043444 and *p* = 0.019994, respectively, *N* = 3), consistent with involvement of Ca^2+^-associated signaling. Surprisingly, cells exposed to 12 h of slow or fast melanopsin stimulation did not show significant *TNF* or *IL8* induction (Figures S3B and S3C). To further characterize the dependence of *IL8* and *TNF* expression on melanopsin stimulation frequency, we stimulated the cells across a wider range of stimulation frequencies. Interestingly, gene expression showed an approximately sigmoidal dependence on melanopsin stimulation frequency, with a clear tendency toward saturation at higher stimulation frequencies (Figure 2E).

### Effects of temporal stimulation patterns on *TNF* and *IL8* expression

To investigate whether the temporal regularity of melanopsin stimulation influenced *IL8* and *TNF* expression, cells were stimulated using a temporally irregular light-pulse sequence. Cells were exposed to 55 light pulses distributed irregularly over 1 h (Figure 3Ai, lower trace). Of the inter-stimulus intervals, 36% were 60 s or shorter (Figure 3B red region). Intriguingly, the gene induction was significantly weaker with the random stimulation regime compared to the regular (*p* = 0.022434 and *p* = 0.010417, *N* = 3; Figure 3C). Next, we compared stimulation regimes with the same number of light pulses but different stimulation frequencies and durations. Thus, cells were either stimulated every 2 min for 1 h (30 pulses, corresponding to 8 mHz) or every minute for half an hour (30 pulses, corresponding to 15 mHz) followed by half an hour without any stimulation (Figure 3Aii, upper and lower trace, respectively). *TNF* induction was significantly lower following the slower stimulation regime (*p* = 0.047527, *N* = 3), whereas *IL8* expression did not differ significantly between the two regimes (*p* = 0.420798, *N* = 3 Figure 3D). Together, these results demonstrate that *TNF* and *IL8* expression is sensitive to the temporal pattern of melanopsin stimulation, although the two genes show distinct responses to stimulation frequency and timing.

**Figure 3:**
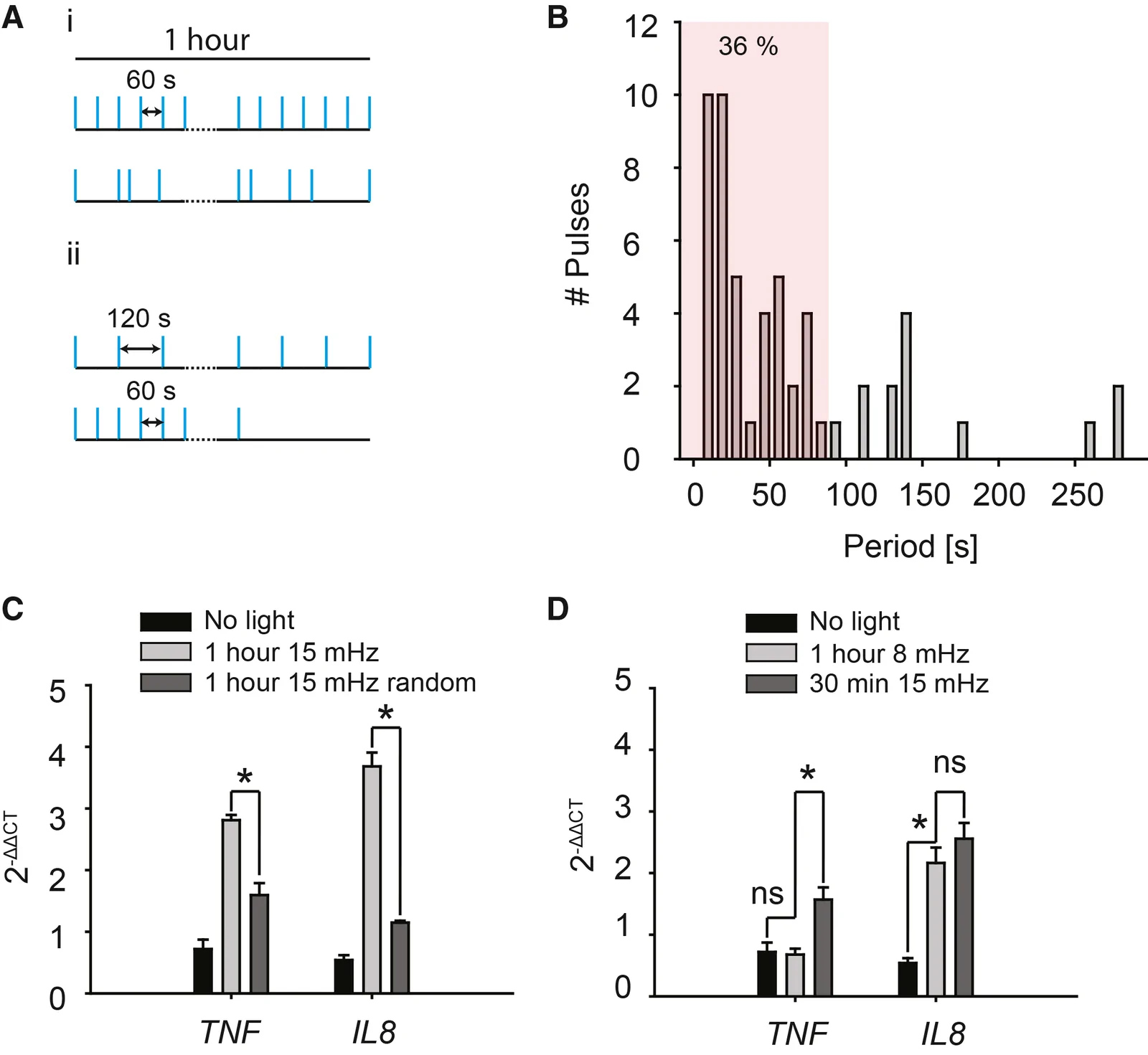
*TNF* and *IL8* expression differ between temporal melanopsin stimulation patterns (A) Schematic of the two different light stimulation paradigms: (i) random stimulation and (ii) equal numbers of light pulses delivered at different stimulation frequencies and durations. (B) The distribution of pause time in-between light pulses for paradigm (i). Red region denotes pauses less than 60 s. (C) The induction of *TNF* and *IL8* is lower for the random stimulation paradigm (i). (D) The induction of *TNF*, but not *IL8*, is lower for stimulation paradigm (ii). *p* values were calculated with one-way or two-way ANOVA followed by Tukey’s post hoc multiple-comparison test; average and SEM for *n* = 3 for independent cultures. ∗ *p* <0.05 and ∗∗ *p* <0.01.

### Transcriptomic responses to different melanopsin stimulation frequencies

Next, we investigated transcriptional responses to different temporal melanopsin stimulation regimes in an unbiased fashion. To assess transcriptomic responses to different melanopsin stimulation frequencies, we stimulated cells for either 1 or 12 h with light pulses delivered every minute (∼15 mHz stimulation) or every 2 min (∼8 mHz stimulation). After the experiment, cells were lysed and prepared for RNA sequencing. In total, 32 bulk samples (around 500 cells in each) were multiplexed and sequenced with molecular counting.^32^ To control for any non-specific or toxic effects of either light itself or the heterologous expression, analyses controlled for non-stimulated cells as well as cells only expressing empty vector. Directly comparing cells expressing empty vector with or without light stimulation did not result in any significantly differentially expressed genes (DEGs) (data not shown).

Principal-component analysis (PCA) revealed distinct clustering of samples by cell type (cells expressing melanopsin vs*.* empty vector), with PC1 and PC2 accounting for 84% and 5% of the variance, respectively (Figure S4A). The separation along PC1 indicates clear transcriptional differences between the two groups. Also considering the type of stimulation (low and high frequency for 1 or 12 h) reveals clustering as well (Figure S4B). To assess the relationship between the principal components (PCs) and the Cell_type variable (melanopsin vs. empty), Kruskal-Wallis tests were performed. A significant difference was found in PC1 (χ^2^ = 21.05, *p* = 4.47 × 10^−6^) and PC4 (χ^2^ = 7.29, *p* = 0.006933) but not in PC2, PC3, or PC5 (*p* > 0.05). Assessing the relationship between the PCs and the stimulation variable (five states) showed a significant association between PC2 and stimulation (χ^2^ = 15.7, *p* = 0.0034), while PC1, PC3, PC4, and PC5 showed no significant differences across the stimulation conditions (*p* > 0.05) (data not shown).

The RNA sequencing analysis identified 215 DEGs between the fast (15 mHz) and slow (8 mHz) melanopsin stimulation regimes after 1 h of stimulation, with “8 mHz” serving as the reference level (Figure 4A; Table S1). Of these, 131 genes were over-expressed, and 84 were under-expressed in the fast stimulation regime relative to the slow regime. After 12 h of stimulation, 27 DEGs (17 over-expressed and 10 under-expressed) were identified between the fast and slow stimulation regime, with the slow (8 mHz) condition again serving as the reference (Figure S5A; Table S2). Of note, there was no overlap between DEGs of 1 hour and 12 h of stimulation, demonstrating distinct transcriptional endpoint profiles after 1 and 12 h of stimulation. To determine whether transcriptional differences between the fast and slow stimulation regimes were specifically associated with melanopsin expression, we additionally performed an interaction-model analysis testing whether the fast-versus-slow response differed between melanopsin and empty control cells. At 1 h, this analysis identified 78 genes exhibiting significant cell-type-dependent differences between the fast and slow stimulation regimes (Table S3), including 23 genes overlapping with the additive-model DEGs. These overlapping genes represent higher-confidence candidates for melanopsin-dependent transcriptional responses to the temporal stimulation regime, whereas the additive-model DEGs more broadly represent transcriptional responses associated with the fast-versus-slow stimulation comparison. In contrast, although nominal interaction-level signals were detectable at 12 h, no genes survived transcriptome-wide multiple-testing correction. Given that persistence of melanopsin-evoked signaling was not established over the full 12-h stimulation period, the mechanistic basis of this difference cannot be determined from the present data.

**Figure 4:**
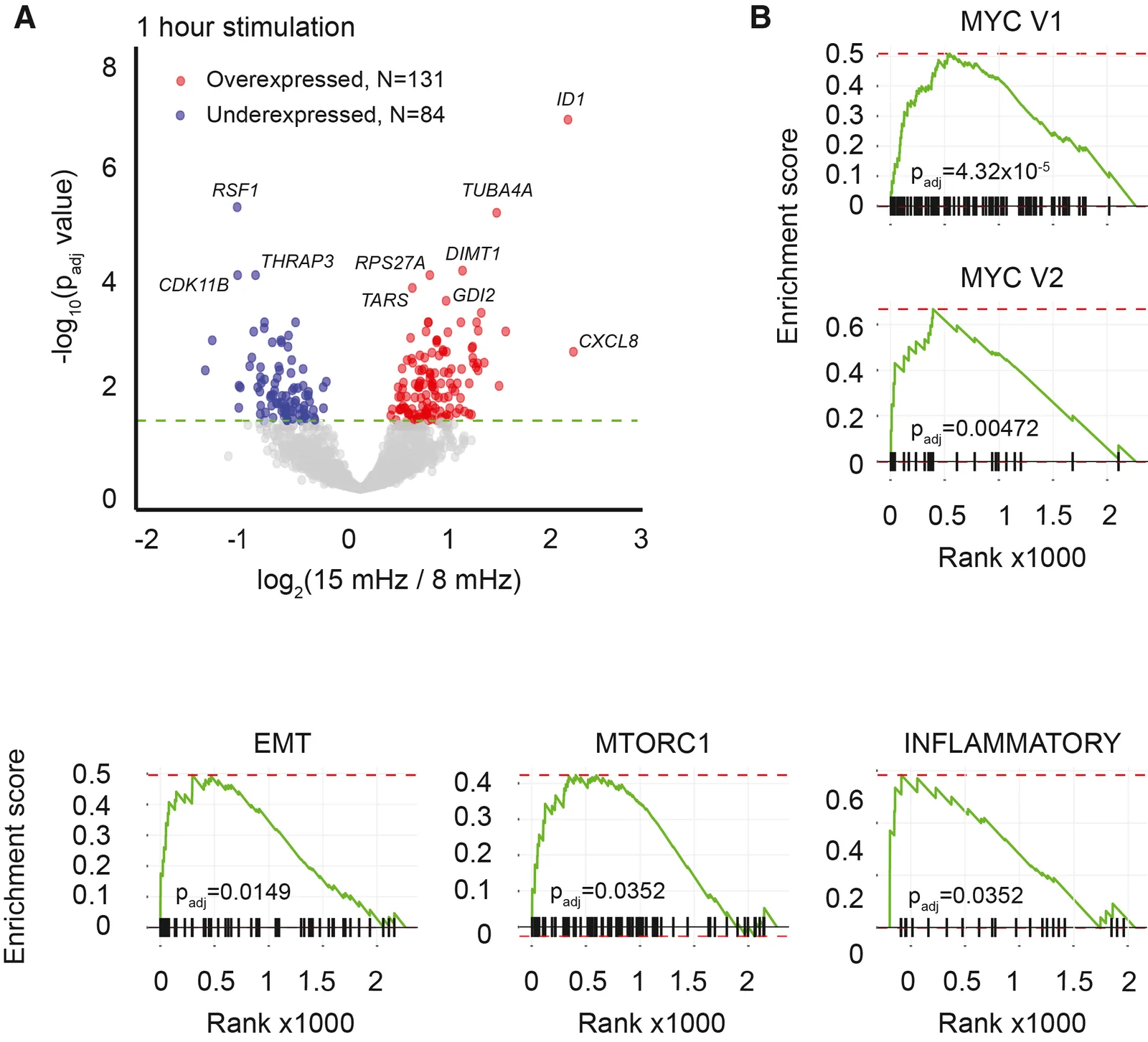
Transcriptomic responses to fast and slow melanopsin stimulation after 1 h (A) Volcano plot of bulk RNA-seq data for 1 h of stimulation showing the log_2_ fold change (*x* axis) against the -log_10_ adjusted *p* value (*y* axis). Genes with adjusted *p* value < 0.05 are highlighted: over-expressed genes are marked in red, and under-expressed genes are marked in blue. Non-significant genes are shown in gray. *n* = 3 for independent biological replicates (cultures) per condition. (B) Enrichment plots of the gene set enrichment analysis results of transcriptomic data. Enrichment plots display the significant pathways identified by gene set enrichment analysis (GSEA) for 1 h using the Hallmark gene sets from MSigDB. The *x* axis represents the rank order of genes in the list, while the *y* axis indicates the running enrichment score. Tick marks along the *x* axis denote the positions of genes from the respective gene set in the ranked list. Pathways with false discovery rate (FDR) < 0.05 were considered significantly enriched. See also Figure S5.

Functional enrichment analysis of the 1-h additive-model DEGs revealed broad enrichment for translation- and ribosome-associated processes, including cytoplasmic translation, ribosome biogenesis, RNA binding, protein binding, and cellular organization terms. To determine whether these functional themes were retained among genes showing melanopsin-dependent responses to the stimulation regime, enrichment analysis was repeated using the 1-h interaction-model DEGs. Despite the smaller and more stringent gene set, the interaction-model DEGs showed enrichment for overlapping biological categories, including structural constituent of ribosome, RNA binding, cytoplasmic translation, ribosomal subunit biogenesis, stress-response regulation, cytosolic ribosome, extracellular exosome, and focal adhesion. These findings suggest that translational/ribosomal and cellular organization programs are associated with the early response to the different temporal stimulation regimes, with a subset showing melanopsin-dependent effects.

To identify potential regulators of stimulation-associated gene expression changes, we applied ChEA3, a web-based tool for transcription factor enrichment analysis, to DEGs between fast and slow melanopsin stimulation regimes after 1 and 12 h of stimulation.^33^ The top 10 enriched transcription factors for each time point are listed in Table 1 and shown in Figures S6A and S6B, respectively.

**Table 1:** Top 10 (based on MeanRank) transcription factors predicted by ChEA3 enrichment analysis

Experimental condition	Rank	TF	MeanRank	Overlapping genes
1 h	1	PA2G4	7.5	28
	2	ZNF888	13	13
	3	ZNF146	38.33	44
	4	POU5F1B	46	22
	5	ZNF207	48.5	56
	6	BPTF	50.5	23
	7	PRMT3	53	33
	8	MYC	53.17	116
	9	HIF1A	56.4	53
	10	CENPA	62	15
12 h	1	RFX8	9.5	6
	2	ZNF469	10.5	6
	3	FOXD1	10.5	6
	4	PRRX1	11.67	8
	5	TWIST2	12.5	6
	6	TWIST1	15.25	9
	7	PRRX2	30.67	5
	8	HIF1A	45.4	11
	9	OSR2	51	5
	10	FOXL1	62.33	6

MeanRank, average rank across all transcription factor-target gene libraries. TF, transcription factor

Subsequently, transcription factor (TF)-gene regulatory networks were constructed for both 1- and 12-h stimulation conditions. The 1-h network consists of 169 nodes (10 TFs and 159 genes) and 403 edges (Figure 5) and is available on NDEx: TF_Gene_Regulatory_Network_1h. MYC-associated target enrichment encompassed the highest number of genes (*n* = 116), followed by ZNF207 (*n* = 56) and HIF1A (*n* = 53). Similarly, the 12-h network comprises 26 nodes (10 TFs and 16 genes) and 68 edges (Figure S5B) and is accessible on NDEx: TF_Gene_Regulatory_Network_12h. For 12 h, HIF1A-associated target enrichment encompassed the highest number of genes (*n* = 11).

**Figure 5:**
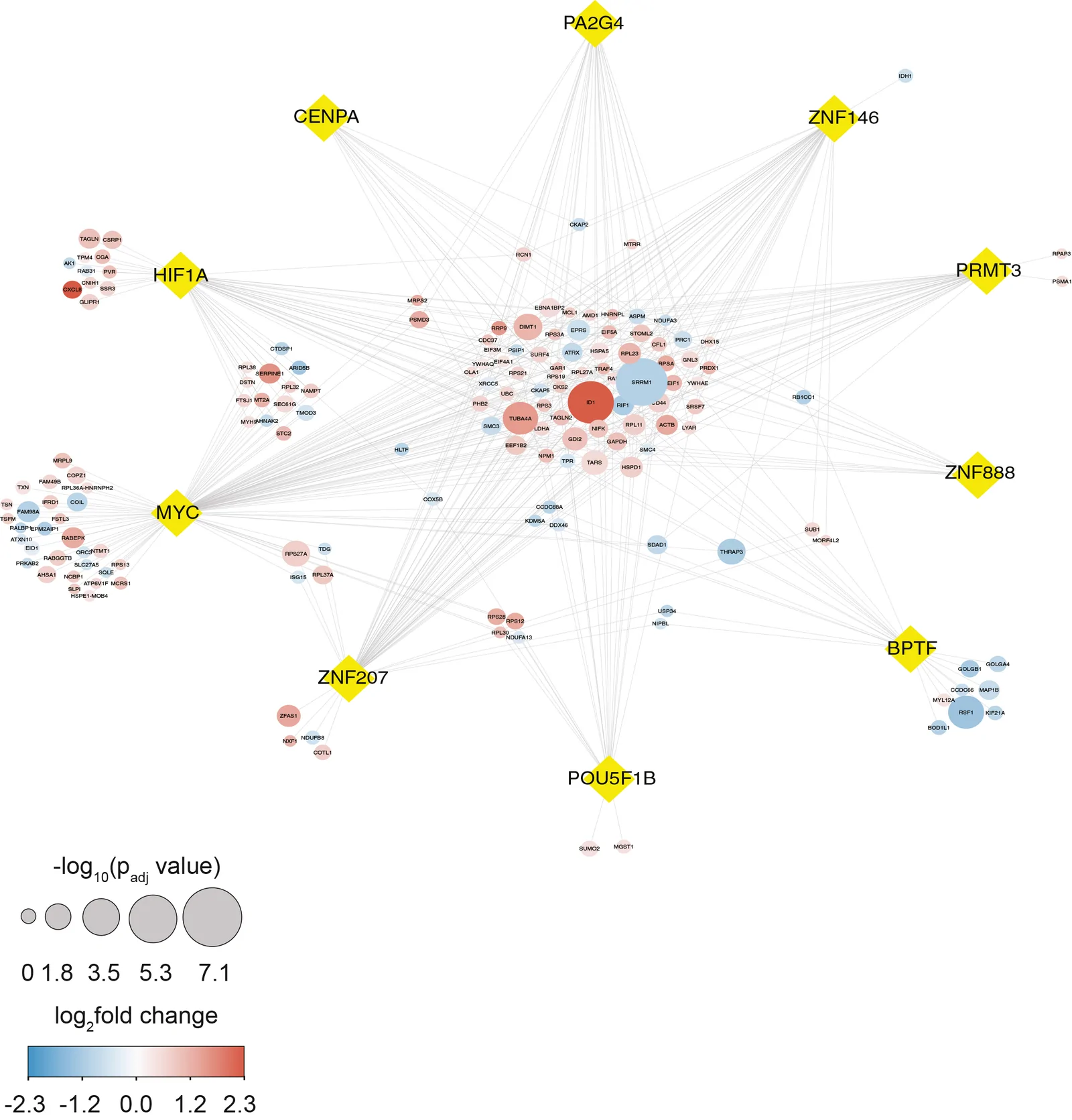
Transcription factor-gene regulatory network following 1 h of melanopsin stimulation Transcription factor (TF)-gene regulatory network constructed using differentially expressed genes (DEGs) after 1 h of stimulation under fast versus slow melanopsin stimulation regimes. Regulatory interactions are computationally inferred using ChEA3. TFs are represented as yellow diamond-shaped nodes, while target genes are shown as circles. Node size corresponds to -log_10_ adjusted *p* value, with color representing log_2_ fold change (log_2_FC): red for upregulated genes and blue for downregulated genes. Edges indicate regulatory interactions between TFs and their target genes. Among the inferred TF-associated networks, MYC was associated with the largest number of target genes (*n* = 116), followed by ZNF207 (*n* = 56) and HIF1A (*n* = 53). The complete network is available through NDEx for interactive exploration of individual nodes and interactions. See also Figures S5 and S6.

Gene set enrichment analysis was performed to identify significantly enriched pathways associated with the transcriptomic profiles of fast and slow melanopsin stimulation regimes at 1 and 12 h, when taking all genes into account. The ranked gene list derived from DESeq2 was analyzed using the Hallmark gene sets from MSigDB. For 1-h stimulation, GSEA identified significant enrichment of pathways HALLMARK MYC TARGETS V1 (MYC V1), HALLMARK MYC TARGETS V2 (MYC V2), HALLMARK EPITHELIAL MESENCHYMAL TRANSITION (EMT), HALLMARK INFLAMMATORY RESPONSE (INFLAMMATORY), and HALLMARK MTORC1 SIGNALING (MTORC1) (Figure 4B). For 12 h stimulation, GSEA revealed significant enrichment of pathways HALLMARK_CHOLESTEROL HOMEOSTASIS (CHOLESTEROL) and HALLMARK APICAL JUNCTION (Figure S5C). Also, when performing gene set enrichment analysis, there was no overlap in the results between 1 and 12 h of stimulation.

### Proteomic and phosphoproteomic responses to different stimulation regimes

Next, we assessed proteomic and phosphoproteomic responses to the fast and slow melanopsin stimulation regimes. Cells were stimulated for 1 h with light pulses in a similar fashion as for the other experiments and were prepared for phosphopeptide enrichment and mass spectrometry (see STAR Methods for details). To control for any non-specific or toxic effects of either light itself or the overexpression, analyses controlled for non-stimulated cells as well as cells only expressing empty vector. Also, phosphopeptide values were normalized to peptide levels to control for any up- or downregulation at protein level.

### Differential abundance analysis of fast vs. slow stimulation regimes

Proteomic analysis identified 641 differentially abundant proteins (DAPs) between the fast (15 mHz) and slow (8 mHz) melanopsin stimulation regime after 1 h of stimulation, with the slow regime serving as the reference (Table S4). Of these, 193 proteins were upregulated and 448 were downregulated in the fast stimulation regime relative to the slow regime (Figure 6A). In the phosphoproteomics dataset (normalized to protein abundance), 211 differentially abundant phosphopeptides (DAPPs) were detected (Table S5), comprising 109 up- and 102 down-phosphorylated peptides, again using the slow (8 mHz) stimulation regime as the reference (Figure 6B). These peptides corresponded to 92 unique protein identifiers in the up-phosphorylated group and 87 unique protein identifiers in the down-phosphorylated group.

**Figure 6:**
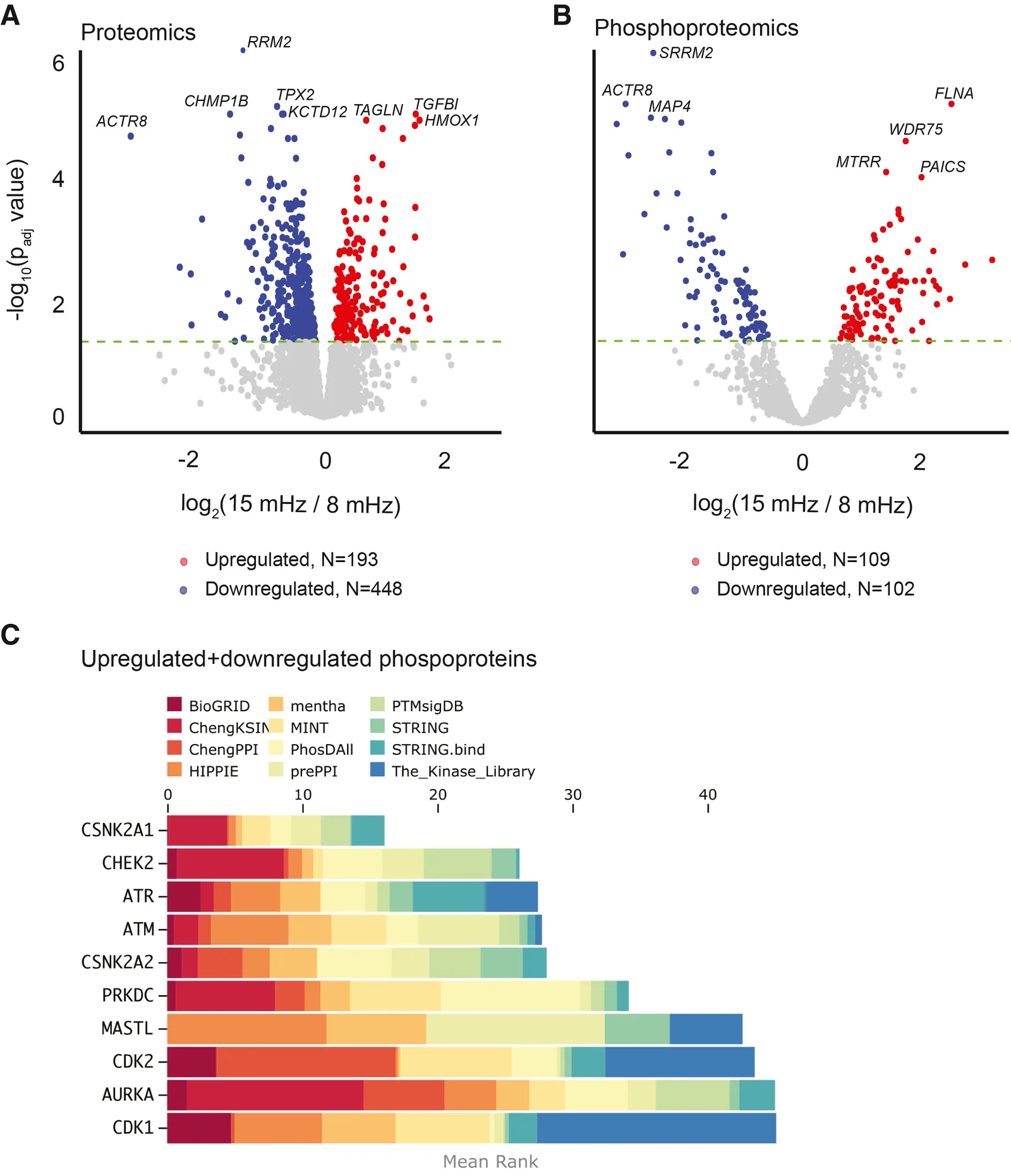
Proteomic and phosphoproteomic responses to fast and slow melanopsin stimulation (A and B) Volcano plots for differential abundance analysis of proteomics and phosphoproteomics at 1 h post-stimulation. Volcano plots for proteomics (A) and phosphoproteomics (B) showing the log2 fold change (*x* axis) against the -log10 adjusted *p* value (*y* axis). Proteins/phosphopeptides with an adjusted *p* value < 0.05 are highlighted: upregulated species are shown in red, downregulated species in blue, and non-significant ones in gray. (C) Weighted library contribution to integrated MeanRank kinase ranks. The bar chart showing the cumulative weighted mean rank of the top 10 kinases. Kinases shown represent computationally predicted upstream regulators identified using KEA3 of differentially abundant phosphoproteins. The *x* axis represents the cumulative weighted mean kinase rank, while the *y* axis lists the top-ranked kinases. The colored segments indicate the contribution of different evidence sources to each kinase ranking. Proteomic and phosphoproteomic analyses were performed using independent biological replicates (*n* = 2–3 per condition).

PCA of the proteomics data using PC1 and PC2 showed limited separation between samples (Figure S4C); however, inclusion of PC3 improved clustering by cell type (melanopsin vs. empty vector), capturing additional variance not explained by the first two components (data not shown). In contrast, PCA of the phosphoproteomics data showed no clear separation between melanopsin and empty vector samples when using PC1 and PC2 (Figure S4D). Inclusion of PC3 in a three-dimensional PCA plot modestly improved the visual distinction between groups, although substantial overlap remained, suggesting that the major sources of variance in the phosphoproteomics dataset are not solely driven by differences in cell type (data not shown).

### Functional enrichment analysis of differentially abundant proteins

GO and KEGG pathway functional enrichment analyses of DAPs were performed using the g:Profiler tool. The top three most significant GO MF terms were “protein binding” (GO:0005515), “catalytic activity” (GO:0003824), and “ATP-dependent activity” (GO:0140657). Among GO BP terms, the most significant were mitotic cell-cycle process (GO:1903047), organelle organization (GO:0006996), and small molecule metabolic process (GO:0044281). The top three GO CC terms included cytoplasm (GO:0005737), chromosome passenger complex (GO:0032133), and phagocytic vesicle (GO:0045335).

### Kinase enrichment analysis reveals potential regulators of phosphorylation changes

To identify candidate upstream kinases potentially associated with the observed phosphopeptide abundance, the sets of corresponding proteins were converted to HGNC gene symbols and submitted to KEA3. The top 10 enriched kinases for up- and down-phosphorylated proteins are listed in Table 2 and shown in Figure 6C.

**Table 2:** Top 10 (based on MeanRank) kinases identified by KEA3 kinase enrichment analysis

Protein set	Rank	Kinase	MeanRank	Overlapping proteins
Differentially abundant phosphoproteins	1	CSNK2A1	16.09	115
	2	CHEK2	26.09	64
	3	ATR	27.45	89
	4	ATM	27.75	108
	5	CSNK2A2	28.1	68
	6	PRKDC	34.18	73
	7	MASTL	42.6	63
	8	CDK2	43.5	121
	9	AURKA	45	65
	10	CDK1	45.09	121

MeanRank, average rank of a kinase across all libraries in which it is present.

### Connecting transcriptomic results with phosphoproteomics

To explore potential regulatory relationships connecting the transcriptomic and phosphoproteomic responses, we integrated the two datasets using transcription factor and kinase enrichment analyses. First, proteins potentially interacting with inferred transcription factors were identified. In total, 103 interactors met the minimum criteria and were retained. Of these, 95 were detected on RNA level in the RNA sequencing experiment. This list was used as input to KEA3, and the top 10 predicted kinases were retrieved (Table 3). Of note, 5 out of 10 predicted kinases were shared between interactor-enriched kinases and those kinases predicted for differentially abundant phosphoproteins: PRKDC (DNA-dependent protein kinase catalytic subunit), CHEK2 (checkpoint kinase 2), ATM, ATR (serine/threonine-protein kinase ATM and ATR), and AURKA (aurora kinase A). The network represents candidate regulatory relationships and should not be interpreted as evidence of direct kinase activation. The network consists of 109 nodes (8 TFs, 91 interactors, and 10 predicted kinases) and 1,006 edges (Figure 7). The network is also available on NDEx: Expression-Kinases regulatory network. It should be noted that TFs and interactors without direct or indirect connections to kinases were excluded from the network.

**Table 3:** Top 10 kinases predicted by KEA3 analysis of transcription factor interactors

Protein set	Rank	Kinase	MeanRank	Overlapping proteins
Transcription factor interactors	1	**PRKDC**	15.36	80
	2	CHEK2	18	80
	3	**ATM**	18.83	87
	4	PLK1	19	80
	5	MAPK8	19.09	84
	6	CHEK1	19.73	75
	7	**ATR**	20.45	78
	8	ABL1	21.36	81
	9	MAPK1	26.36	88
	10	**AURKA**	29.27	75

MeanRank, average rank of a kinase across all libraries in which it is present. Kinases shown in bold were identified both in the transcription factor interactor analysis and in the phosphoproteomic kinase enrichment analysis.

**Figure 7:**
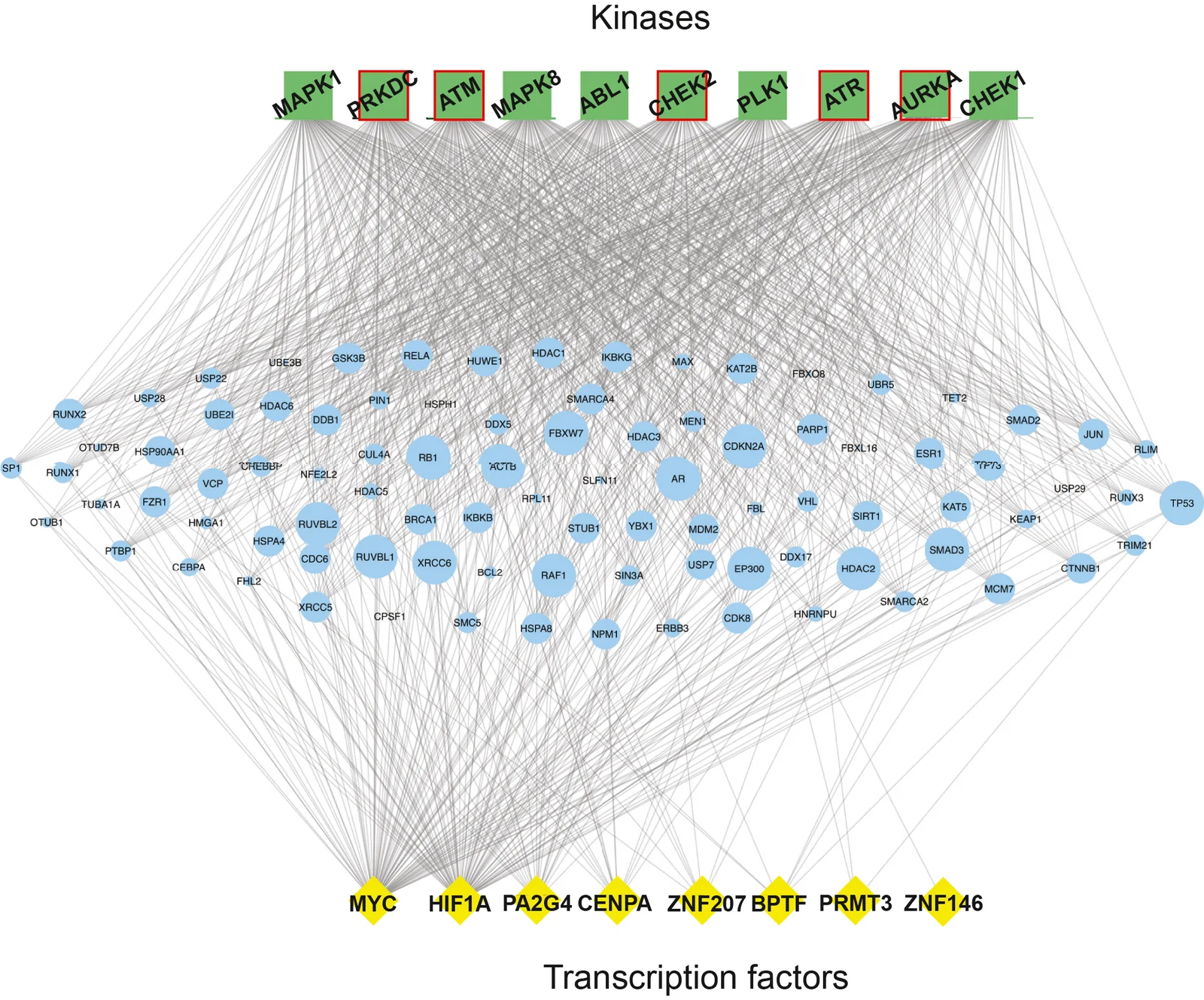
Integrated transcription factor-interactor-kinase regulatory network Expression-kinase regulatory network. The network represents candidate-inferred regulatory relationships between transcription factors (TFs), interactors, and kinases and does not imply direct experimental validation. The network illustrates interactions. Kinases are shown as green rectangles (top), TFs as yellow diamond-shaped nodes (bottom), and intermediate proteins/transcription factor interactors as blue ovals (middle). The size of interactor nodes is scaled according to their degree (number of connections). Edges indicate regulatory/physical interactions between TFs, interactors, and kinases. Kinases with red outlines were independently identified by both inference approaches, i.e., enrichment analysis of TF-associated interactors and enrichment analysis of differentially abundant phosphoproteins. These overlapping candidate kinases comprise PRKDC, CHEK2, ATM, ATR, and AURKA and represent the principal convergent feature highlighted in the network. The complete network is available through NDEx for interactive exploration of individual nodes and interactions. See also Figure S6.

## Discussion

Here, we investigated how intracellular signaling networks respond to distinct temporal patterns of melanopsin stimulation that evoke oscillatory Ca^2+^-associated signaling. By applying stimulation regimes that differed in frequency and temporal regularity, we were able to (1) characterize NF-κB-associated transcriptional responses of *TNF* and *IL8* and (2) perform an unbiased screen of transcriptional and phosphoproteomic responses associated with different temporal stimulation regimes.

Targeted studies of NF-κB using luciferase reporter and RT-qPCR revealed that both *TNF* and *IL8* expression differed across temporal melanopsin stimulation regimes. Regular stimulation produced stronger *TNF* and *IL8* induction than the temporally irregular paradigm, despite similar numbers of light stimuli. However, because intracellular Ca^2+^ dynamics were not directly quantified under the regular and irregular paradigms, these experiments do not establish that cumulative Ca^2+^ activity was equivalent or that periodicity itself was isolated as the causal variable. The matched-pulse experiment further revealed gene-specific temporal sensitivity, with *TNF*, but not *IL8*, differing significantly between the two stimulation regimes. RNA sequencing following 1 h of stimulation revealed an MYC-associated transcriptional signature, supported by both GSEA and ChEA3 analyses. The additive model identified broader transcriptional differences associated with the fast-versus-slow stimulation comparison, whereas the interaction model identified a more stringent subset whose response depended on melanopsin expression. At 1 h, 78 genes showed a significant interaction effect, including 23 genes that overlapped with the additive-model DEGs. We therefore interpret the interaction-model genes as higher-confidence candidates for melanopsin-dependent responses to the temporal stimulation regime, while the broader additive-model signature is interpreted more cautiously as stimulation-associated. MYC, a bHLH-LZ transcription factor, regulates gene networks controlling proliferation and apoptosis, and its dysregulation underlies many cancers, including cervical cancer (from which HeLa cells originate^34^). While *MYC* mRNA levels were not altered between slow and fast melanopsin stimulation, its downstream transcriptional network was differentially regulated: within the broader additive-model transcriptional signature, 116 of the 215 DEGs were potential MYC targets. This raises the possibility that oscillatory signaling may influence MYC-associated transcriptional programs through post-transcriptional mechanisms.

In contrast, 12-h stimulation yielded fewer differentially expressed genes. Because persistence of melanopsin-evoked signaling was not quantified over the full 12-h stimulation period, this later endpoint cannot distinguish downstream biological adaptation from changes or deterioration in the imposed signaling regime and is therefore interpreted descriptively.

Transcriptional responses to temporally patterned stimulation arise within interconnected signaling pathways that include kinase-mediated protein phosphorylation. To explore candidate kinase networks associated with the observed responses, we measured phosphopeptide abundance using label-free mass spectrometry and performed kinase enrichment analysis. Given the relatively limited PCA separation and the use of missing-value imputation, phosphoproteomic findings should primarily be interpreted as hypothesis-generating and inference-based. In parallel, we analyzed DEGs from RNA sequencing to predict upstream transcription factors and their interacting proteins and used these predictions to infer upstream kinases. Notably, the top-ranked kinases from these two inference strategies showed substantial overlap (50% when comparing the top 10 from each method). Among the kinases inferred from our analyses—PRKDC, CHEK2, ATM, ATR, and AURKA—a unifying feature is their central role in the DNA damage response and cell-cycle checkpoint control. These pathways safeguard genome integrity under conditions of replication stress and thereby sustain cellular proliferation. ATM is canonically activated upon DNA double-strand breaks and other threats to DNA integrity and phosphorylates key proteins, including CHEK2, to inhibit cell-cycle progression or induce apoptosis.^35^^,^^36^ PRKDC belongs to the same superfamily of phosphatidylinositol-3-kinase-related kinases as ATM and is involved in the repair of DNA double-strand breaks.^37^ Notably, several of these kinases are connected to MYC biology. Dysregulation of proliferation and genomic instability in cancers with MYC overexpression is counteracted by ATM, and experimental overexpression can activate the ATM DNA damage response pathway.^38^ Similarly, inhibition of PRKDC in MYC-overexpressing human lung fibroblasts is lethal.^39^ This convergence suggests that temporally patterned melanopsin-evoked signaling may intersect with stress-response and checkpoint networks associated with MYC-related transcriptional programs. Although ATM, PRKDC, and CHEK2 are not classical Ca^2+^-dependent kinases, there is growing evidence that ATM activity influences Ca^2+^ handling, including mitochondrial Ca^2+^ uptake.^40^ Because PRKDC and CHEK2 function downstream of ATM-associated DNA damage signaling networks, their enrichment in our analyses identifies these pathways as candidates for further investigation in the context of temporally patterned melanopsin signaling.

Our findings can be considered alongside previous studies that directly controlled intracellular Ca^2+^ waveform parameters. Hannanta-Anan and Chow used melanopsin-based stimulation to characterize Ca^2+^ waveforms with controlled peak amplitudes and duty cycle and found that NFAT transcriptional activity depends more strongly on cumulative Ca^2+^ load than on frequency.^25^ In the present study, the experimentally controlled variable was instead the temporal pattern of melanopsin activation within an intact Gq-coupled signaling network. The regular, irregular, and matched-pulse stimulation paradigms produced distinct downstream transcriptional responses, but the resulting intracellular Ca^2+^ waveforms were not quantitatively matched. Thus, our data do not isolate Ca^2+^ frequency, cumulative Ca^2+^ activity, or periodicity as independent causal variables. Rather, they demonstrate that downstream responses differ between distinct temporal patterns of melanopsin-evoked signaling.

In conclusion, our study identifies broad transcriptional and phosphoproteomic responses associated with distinct temporal patterns of melanopsin stimulation using optogenetics, RNA sequencing, and phosphoproteomics. We provide evidence that NF-κB-associated target gene responses differ between temporal melanopsin stimulation patterns and identify an MYC-associated transcriptional response together with candidate stress-response and checkpoint-associated kinase networks involving PRKDC, CHEK2, and ATM. These findings provide a basis for further investigation of how temporal signaling dynamics shape cellular decision-making, including signal integration, proliferation, and stress responses.

### Limitations of the study

Several limitations of this study should be acknowledged. First, our approach relies on temporally controlled optogenetic activation of melanopsin, which evokes repetitive Ca^2+^ responses within an intact Gq-coupled signaling network. Because intracellular Ca^2+^ waveforms were not quantitatively characterized across all stimulation paradigms, the present experiments cannot determine the individual contributions of Ca^2+^ event frequency, response probability, recovery, duty cycle, or integrated Ca^2+^ activity to the observed downstream responses. Melanopsin is a Gαq-coupled receptor and therefore engages multiple downstream signaling branches, including phospholipase C activation, diacylglycerol production, PKC activation, and potentially MEK/ERK signaling. Accordingly, melanopsin stimulation is expected to generate coordinated oscillatory activity across several signaling nodes rather than a Ca^2+^-exclusive signal. Previous work has shown that PKC activity can oscillate in phase with Ca^2+^ signals and contribute to transcriptional regulation, including NF-κB activation.^16^ Thus, our findings should be interpreted as temporal decoding of melanopsin-evoked signaling within an intact Gq-coupled network, rather than as evidence for decoding of Ca^2+^ oscillation frequency in isolation. Second, we employed HeLa cells, a non-excitable carcinoma-derived line. Ca^2+^ signaling toolkits differ substantially across cell types,^41^ and neuronal and developmental contexts in particular possess specialized Ca^2+^-handling machinery^42^; generalizability to excitable or tissue-specific contexts remains to be established. Third, the use of label-free mass spectrometry reduces sensitivity for phosphopeptide detection and may underestimate the number of proteins contributing to the observed stimulation-associated responses. Finally, owing to experimental complexity, we did not directly quantify the stability of light-evoked Ca^2+^ oscillations over extended stimulation durations, which may contribute to the reduced transcriptional differences observed at later time points.

Relatedly, previous studies from the Lewis laboratory used store-depletion strategies to generate repetitive Ca^2+^ oscillations with minimal upstream receptor signaling, providing a particularly clean framework for isolating Ca^2+^-dependent decoding mechanisms.^13^ In contrast, our receptor-based approach intentionally preserves receptor-proximal signaling complexity, engaging multiple downstream pathways alongside Ca^2+^ dynamics. While this introduces additional pathway engagement, it enables analysis of how temporally patterned melanopsin signaling is associated with interconnected kinase and transcriptional circuits within an intact signaling network. These approaches are therefore complementary, addressing different levels of the frequency-encoding problem.

## Resource availability

### Lead contact

Requests for further information and resources should be directed to and will be fulfilled by the lead contact, Erik Smedler (erik.smedler@gu.se).

### Materials availability

Stable melanopsin-expressing HeLa cell populations and the pLVX-OPN4-IRES-mCherry expression construct generated in this study are available from the lead contact upon reasonable request and with a completed Materials Transfer Agreement.

### Data and code availability

• Bulk RNA sequencing data have been deposited in the NCBI Gene Expression Omnibus (GEO) and are publicly available as of the date of publication under accession number GSE319210.
• Proteomics and phosphoproteomics data have been deposited in the ProteomeXchange Consortium via the PRIDE repository and are publicly available as of the date of publication under accession number PXD074676.
• TF-gene regulatory networks generated in this study have been deposited in the Network Data Exchange (NDEx) repository and are publicly available. Network identifiers are provided below:
  • TF_Gene_Regulatory_Network_1h (NDEx UUID: 5d50323d-ef7a-11ef-b81d-005056ae3c32): https://www.ndexbio.org/viewer/networks/5d50323d-ef7a-11ef-b81d-005056ae3c32
  • TF_Gene_Regulatory_Network_12h (NDEx UUID: 14c37626-f062-11ef-b81d-005056ae3c32): https://www.ndexbio.org/viewer/networks/14c37626-f062-11ef-b81d-005056ae3c32
• The Expression-Kinases regulatory network generated in this study has been deposited in the Network Data Exchange (NDEx) repository and is publicly available:
  • Expression-Kinases regulatory network (NDEx UUID: d4c54cc9-7dc3-11f0-a218-005056ae3c32):
https://www.ndexbio.org/viewer/networks/d4c54cc9-7dc3-11f0-a218-005056ae3c32
• Custom MATLAB and R scripts used for calcium imaging analysis, transcriptomic analysis, and phosphoproteomic analysis are publicly available at GitHub https://github.com/smedler-lab/melanopsin-paper
• Any additional information required to re-analyze the data reported in this paper is available from the lead contact upon request.

## Acknowledgments

We are grateful to Dr. Dmitry Usoskin and Dr. Sten Linnarsson for the previous development of TTL-based control that was utilized in this project; Dr. Haifeng Ye for kindly providing the pIRES2-OPN4AI plasmid; And Dr. Amit Zeisel, Dr. Anna Johnsson, and Dr. Marcela Ferella for valuable help with RNA sequencing. Proteomic analysis was carried out at the Proteomics Karolinska (PK/KI) by Carina Palmberg and Dr. Alexey Chernobrovkin as well as at the Proteomics Core Facility, Sahlgrenska academy, Gothenburg University, with financial support from SciLifeLab and BioMS. This work was supported by the 10.13039/501100004359Swedish Research Council (ES: 2022-02185, PU: 2021-03108, and 2025-01020), the 10.13039/501100002794Swedish Cancer Society ( to P.U., grant 25 4957), Linnaeus Center in Developmental Biology for Regenerative Medicine (DBRM), the 10.13039/501100004063Knut and Alice Wallenberg Foundation (E.S.: Wallenberg Center for Molecular and Translational Medicine at the University of Gothenburg; P.U.: CLICK and Research Fellow), the Karolinska Institute’s KID doctoral program (ES), Åke Wiberg’s Foundation (P.U. and E.S.), 10.13039/501100006285Magnus Bergvall's Foundation (P.U. and E.S.), and 10.13039/501100003186Fredrik and Ingrid Thurings Foundation (P.U. and E.S.).

## Author contributions

Conceptualization, E.S. and P.U.; methodology, E.S., P.N., M.V.-G., and P.U.; investigation, E.S., P.N., M.V.-G., and J.T.; formal analysis, E.S., P.N., M.V.-G., and J.T.; data curation, E.S., P.N., M.V.-G., and J.T.; visualization, E.S. and P.N.; writing – original draft, E.S. and P.N.; writing – review and editing, E.S., P.N., M.V.-G., J.T., and P.U.; supervision, E.S. and P.U.; funding acquisition, E.S. and P.U.; project administration, E.S. and P.U.

## Declaration of interests

The authors have no conflicts of interest to declare.

## Declaration of generative AI and AI-assisted technologies in the manuscript preparation process

During the preparation of this manuscript, the authors used generative AI tools (including ChatGPT and OpenAI) to assist with language editing and refining text. All content generated with AI assistance was critically reviewed, edited, and verified by the authors, who take full responsibility for the accuracy, originality, and integrity of the published work.

## STAR★Methods

### Key resources table

**Table:** 

REAGENT or RESOURCE	SOURCE	IDENTIFIER
**Bacterial and virus strains**		

Lentivirus expressing OPN4-IRES-mCherry	This paper	N/A
Lentivirus expressing empty vector (pLVX-IRES-mCherry)	This paper	N/A

**Chemicals, peptides, and recombinant proteins**		

All-trans retinal (ATR)	Sigma-Aldrich	Cat# R2500
U73122	Sigma-Aldrich	Cat# U6756
2-APB	Sigma-Aldrich	Cat# D9754
SC-514	Sigma-Aldrich	Cat# SML0557
Ro 106-9920	Sigma-Aldrich	Cat# 557550
Fura-2 AM	Thermo Fisher / Molecular Probes	Cat# F1221

**Critical commercial assays**		

RNeasy kit	Qiagen	Cat# 74104
RT2 First Strand Kit	Qiagen	Cat# 330401
RT2 SYBR Green ROX qPCR Mastermix	Qiagen	Cat# 330513
Dual-Luciferase Reporter Assay System	Promega	Cat# E1910
Pierce TiO2 Phosphopeptide Enrichment and Clean-up Kit	Thermo Fisher Scientific	Cat# 88301
Dead Cell Apoptosis Kits with Annexin V for Flow Cytometry	Thermo Fisher Scientific	Cat# V13242

**Deposited data**		

Bulk RNA sequencing data	NCBI Gene Expression Omnibus	GSE319210
Proteomics and phosphoproteomics data	ProteomeXchange/PRIDE	PXD074676
TF-Gene regulatory network (1 h)	NDEx platform	https://www.ndexbio.org/viewer/networks/5d50323d-ef7a-11ef-b81d-005056ae3c32
TF-Gene regulatory network (12 h)	NDEx platform	https://www.ndexbio.org/viewer/networks/14c37626-f062-11ef-b81d-005056ae3c32
Expression-Kinases regulatory network	NDEx platform	https://www.ndexbio.org/viewer/networks/d4c54cc9-7dc3-11f0-a218-005056ae3c32
Custom MATLAB and R analysis scripts	GitHub	https://github.com/smedler-lab/melanopsin-paper

**Experimental models: Cell lines**		

Human: HeLa melanopsin-high stable cell line	This paper	N/A
Human: HeLa melanopsin-low stable cell line	This paper	N/A
Human: HeLa empty-vector stable cell line	This paper	N/A
Human: HeLa cells	ATCC	CCL-2
Human: 293FT cells	Thermo Fisher Scientific	Cat# R70007

**Oligonucleotides**		

*BAX* primers	This paper	See STAR Methods
*BCL2* primers	This paper	See STAR Methods
*CCND1* primers	This paper	See STAR Methods
*FGF8* primers	This paper	See STAR Methods
*GAPDH* primers	This paper	See STAR Methods
*IL8* primers	This paper	See STAR Methods
*MYC* primers	This paper	See STAR Methods
*OPN4* primers	This paper	See STAR Methods
*TNF* primers	This paper	See STAR Methods

**Recombinant DNA**		

pLVX-IRES-mCherry	Clontech	631237
pIRES2-OPN4AI	Ye et al.^43^	Original publication
pLVX-Melanopsin-IRES-mCherry	This paper	N/A
psPAX2 packaging plasmid	Addgene	Plasmid #12260
pMD2.G envelope plasmid	Addgene	Plasmid #12259

**Software and algorithms**		

DESeq2	Love et al.^44^	https://bioconductor.org/packages/DESeq2
g:Profiler	Kolberg et al.^45^	https://biit.cs.ut.ee/gprofiler/gost
ChEA3	Keenan et al.^33^	https://maayanlab.cloud/chea3/
fgsea	Korotkevich et al.^46^	https://bioconductor.org/packages/fgsea
KEA3	Kuleshov et al.^47^	https://maayanlab.cloud/kea3
missForest	Stekhoven and Buhlmann^48^	https://cran.r-project.org/web/packages/missForest/index.html
Proteome Discoverer 3.0	Thermo Fisher Scientific	N/A
limma	Ritchie et al.^49^	https://bioconductor.org/packages/release/bioc/html/limma.html
Cytoscape	Shannon et al.^50^	https://cytoscape.org/
ggplot2	Wickham^51^	https://ggplot2.tidyverse.org/
biomaRt	Durinck et al.^52^	https://bioconductor.org/packages/release/bioc/html/biomaRt.html
BioGRID	Oughtred et al.^53^	https://thebiogrid.org/

### Experimental model and study participant details

#### Cell lines

Human HeLa cells (ATCC, CCL-2) and 293FT cells (Thermo Fisher Scientific, Cat# R70007) were used in this study. HeLa cells are an immortalized cervical carcinoma cell line originally derived from a 31-year-old female patient. 293FT cells are a derivative of the HEK293 cell line, which originated from human embryonic kidney cells derived from a female fetus and was subsequently engineered to stably express the SV40 large T antigen. 293FT cells were used exclusively for lentiviral production, while all optogenetic, imaging, molecular, transcriptomic, and proteomic experiments were performed in HeLa-derived stable cell lines. Cells were maintained at 37°C in a humidified incubator with 5% CO_2_ and cultured in Dulbecco’s Modified Eagle Medium (DMEM; Invitrogen) supplemented with 10% fetal bovine serum (FBS; Invitrogen) and 1% penicillin–streptomycin (Invitrogen). Cells were routinely passaged according to standard cell culture procedures. The cell lines were not independently authenticated in this study. Cell cultures were tested for mycoplasma contamination by Eurofins and were confirmed to be negative. For optogenetic stimulation experiments, HeLa cells were seeded at 100,000 cells per well in six-well plates one day before stimulation and cultured in the presence or absence of all-trans retinal (100 nM), as indicated. No human participants, primary human samples, animals, plants, or microorganisms were used in this study. Therefore, institutional review board approval, informed consent, and animal ethics approval were not required.

#### Sex as a biological variable

All experiments were performed using established female-derived human cell lines. Consequently, the influence of sex on the observed cellular responses could not be evaluated. The findings therefore reflect responses in female-derived cell models, and the potential influence of sex on the reported signaling, transcriptional, and phosphoproteomic responses remains to be determined.

### Method details

#### Generation of stable melanopsin-expressing cell lines

The melanopsin gene (*OPN4*^43^) was cloned from pIRES2-OPN4AI into the pLVX-IRES-mCherry expression vector (Clontech PT5061-5). Lentiviral particles were produced in 293FT cells by co-transfection of pLVX-Melanopsin-IRES-mCherry or empty pLVX-IRES-mCherry vector together with the packaging plasmid psPAX2 (Addgene #12260) and envelope plasmid pMD2.G (Addgene #12259) using the calcium phosphate transfection method. Viral supernatants were collected, concentrated by ultracentrifugation, and used to transduce HeLa cells in the presence of polybrene. Melanopsin-expressing and empty-vector control cells were isolated by fluorescence-activated cell sorting (BD FACSAria III). Stable expression was verified by RT-qPCR and flow cytometry (BD FACSCanto II).

#### Light stimulation

A custom-made light stimulation setup was assembled using the following: a blue (470 nm) Rebel LED pre-mounted on a 10 mm square base (Luxeon Star LEDs, MR-B0030-10S) attached to 25 mm square x 10 mm high alpha heat sink (Luxeon Star LEDs, LPD25-10B) using pre-cut thermal adhesive tape (Luxeon Star LEDs, LXT-R-10). Light was focused with a Carclo 18.4 deg lens (Luxeon Star LEDs, 10412). The driving voltage was applied with a power-source (TTi, PL303QMD) connected to a relay (RS, 632-089) and 700 mA constant current LED driver (Recom, RCD-24-0.70/W). The relay was controlled by a DAQ based system (Measurement Computing, USB-1208FS-PLUS) and custom-made software. Light intensity was measured with a FieldMaxII-TO power meter (Coherent). For studying Ca^2+^ dynamics, 5 V was used and for long-term downstream studies 4.3 V was used to reduce light toxicity.

#### Calcium imaging

Cells were loaded with 5 μM Fura-2/AM (Molecular Probes) in normal culture medium at 37°C for 25 minutes. Then, the cells were washed and immersed in 2 mL of Krebs-Ringer’s medium (in mM): 119.0 NaCl, 2.5 KCl, 2.5 CaCl_2_, 1.3 MgCl_2_, 1.0 NaH_2_PO_4_, 20.0 HEPES (pH 7.4) and 11.0 dextrose. The Ca^2+^ recording experiments were performed using a temperature-controlled chamber (QE-1; Warner Instruments) and a cooled electron-multiplying charged-coupled camera (Quant EM 512 SC, Photometrics) mounted on an upright fixed stage microscope (AxioExaminer.A1, Carl Zeiss) equipped with a 20×/1.0NA objective (Carl Zeiss). Excitation at 340 and 380 nm was controlled with an illumination system (DG4, Sutter Instrument). MetaFluor (Molecular Devices) was used to control all devices. Fura-2 ratio traces were normalized to baseline and expressed as R/R_0_, where R_0_ was defined as the mean F340/F380 ratio during the first 10 s of recording, except where otherwise specified for response analysis. Drugs were bath applied.

#### Response analysis

To robustly estimate baseline during prolonged recordings with slow baseline drift, a time-varying baseline R_0_ (t) was calculated using a rolling 0.1 quantile of the ratio signal. Specifically, for each time point *t*, baseline was defined as the 10th percentile of R values within a ±40 s window around *t*. At the beginning and end of recordings, forward and backward filling was used to ensure a full-length baseline estimate.

#### Calcium peak detection

Calcium responses were analyzed using ΔR/R_0_. Local maxima were identified as candidate peaks when ΔR/R_0_ at time *t* exceeded adjacent values. Candidate peaks were filtered based on prominence and inter-peak distance. Prominence was defined as the difference between the peak amplitude and the minimum ΔR/R_0_ within a ±45 s window surrounding the peak. Peaks with prominence greater than six times the median absolute deviation of the full trace were retained. Peaks occurring within 20 s of each other were filtered by retaining only the peak with the higher prominence.

#### Response rate analysis

To determine whether a given light stimulus evoked a Ca^2+^ response, peaks were detected within a 30 s window following stimulus onset. A detected peak within this window was classified as a response. Response rate was calculated as the fraction of stimuli that elicited a detectable Ca^2+^ peak.

#### Peak width analysis

Peak width was defined as the time from onset of the 5 s light stimulus to the point at which ΔR/R_0_ returned to within 10% above baseline. Baseline for width estimation was calculated as the mean ΔR/R_0_ between 30 s and 50 s after stimulation.

#### RT-qPCR

RNA was harvested using the RNeasy kit (Qiagen) and cDNA was synthesized using the RT^2^ First strand kit (Qiagen). qPCR was performed on an Applied Biosystems qPCR machine using the RT^2^ SYBR Green ROX qPCR Mastermix (Qiagen). Analysis was performed according to the ΔΔ CT-method.^54^ The following primers were used. *OPN4* CCCCTGCTCATCATCATCTTCTG (forward, Tm 67°C) TGACAATCAGTGCGACCTTGGC (reverse, Tm 70°C); *IL8* ACTGAGAGTGATTGAGAGTGGAC (forward, Tm 61°C) AACCCTCTGCACCCAGTTTTC (reverse, Tm 63°C); *CCND1* TGGAGCCCGTGAAAAAGAGC (forward, Tm 68°C) TCTCCTTCATCTTAGAGGCCAC (reverse, Tm 66°C); *FGF8* GACCCCTTCGCAAAGCTCAT (forward, Tm 68°C) CCGTTGCTCTTGGCGATCA (reverse, Tm 68°C); *BAX* CCCGAGAGGTCTTTTTCCGAG (forward, Tm 68°C) CCAGCCCATGATGGTTCTGAT (reverse, Tm 67°C); *BCL2* GGTGGGGTCATGTGTGTGG (forward, Tm 69°C) CGGTTCAGGTACTCAGTCATCC (reverse, Tm 67°C); *TNF* GAGGCCAAGCCCTGGTATG (forward, Tm 69°C) CGGGCCGATTGATCTCAGC (reverse, Tm 69°C); *MYC* GGCTCCTGGCAAAAGGTCA (forward, Tm 68°C) CTGCGTAGTTGTGCTGATGT (reverse, Tm 65°C) *GAPDH* ACAACTTTGGTATCGTGGAAGG (forward, Tm 65°C) GCCATCACGCCACAGTTTC (reverse, Tm 67°C). Expected amplicon sizes were determined by *in silico* mapping of primer sequences to human reference cDNA transcripts (RefSeq). Primer binding sites were identified for each forward primer and the reverse-complement of each reverse primer, and amplicon length was calculated as the number of nucleotides between these positions, inclusive of the primers. qPCR products for *OPN4*, with *GAPDH* as reference control, were resolved on a 1% agarose gel.

#### Luciferase reporter assay

Cells were transiently co-transfected using Effectene (Qiagen) with plasmids carrying NF-κB luciferase reporter and Renilla 24 h before experiments. Thereafter, cells were harvested, and luciferase activity was measured using the dual-luciferase reporter assay system (Promega).

#### Cell viability assay

Possible toxic effects of light stimulation were assayed using the Annexin V kit (ThermoFisher). Stained cells were analyzed by cytometry (BD FACSCanto II). Positive controls were cells treated with 1 μM staurosporine for 19 h.

#### Bulk RNA sequencing

##### Sample preparation

Cells in six multi-well dishes were washed and enzymatically dissociated in pre-warmed TrypLE Express (Invitrogen). Next, they were counted and diluted to 500 cells/μL and kept on ice all the time. After this, 1 μL of cell suspension was mixed with 1.8 μL of lysis mix with beads (0.09 % Triton X-100, nuclease free water, 11.25 mM DTT, 2.25 mM dNTP and 2 units TaKaRa RNase inhibitor) and incubated in 37°C for 10 min. C1 beads (Invitrogen) were prepared to carry C1-T1-T31. Then, the suspension was mixed and incubated in 72°C for 3 min and cooled to 4°C. Immediately after, 3.6 μL of RT mix (0.98x SuperScript first strand buffer, 6 mM MgCl_2_, 0.81 M Betaine, nuclease free water, 1.6 units TaKaRa RNase inhibitor, 18 units SuperScript II and 2.04 μM C1-P1-RNA-TSO6) was added and incubated in 42°C for 90 min. Then, beads were bond on a magnet and supernatant discarded. 10 μL of PCR mix (111 mM dNTP, 0.48 μM C1-P1-PCR2, nuclease free water and 1x KAPA HiFi hot start ready mix) was added and beads were re-suspended. At last, PCR was run (95°C for 3 min; 6 cycles of 98°C 20 s, 62°C 4 min, 72°C 6 min; 10 cycles of 98°C 20 s, 68°C 30 s, 72°C 6 min; 8 cycles of 98°C 30 s, 68°C 30 s, 72°C 7 min, and 72°C for 10 min) and supernatant saved. The concentration and quality of cDNA were measured using the Quant-iT assay (Invitrogen) and BioAnalyzer (Agilent Technologies), respectively.

##### Library preparation and sequencing

A sequencing library for Illumina HiSeq2000 was prepared using Tn5 transposase. cDNA library was cleaned with AMPure (Beckamn Coulter) and quantified with qPCR. All 32 samples were pooled together and sequenced in one lane.

##### Differential gene expression analysis

Differential gene expression analysis was conducted using the DESeq2 package in R.^44^ A DESeqDataSet object was created from a count matrix and sample metadata with a design formula (∼ Cell type + Stimulation) to account for the effects of cell type and stimulation in the analysis. By stimulation, we mean exposing cells for one hour or 12 h to melanopsin-activating light pulses delivered every minute (15 mHz) or every second minute (8 mHz). Normalization and differential expression testing were performed, and differentially expressed genes (DEGs) were identified with a false discovery rate (FDR) threshold of < 0.05. To assess whether frequency-dependent transcriptional responses differed between melanopsin-expressing and Empty control cells, an additional interaction-model analysis was performed using the design formula (∼ Cell_type ∗ Stimulation). Interaction contrasts were used to test whether the transcriptional response to fast versus slow melanopsin stimulation differed between Melanopsin and Empty cells at each time point (1 h and 12 h).

##### Principal component analysis

To assess overall variability among samples, principal component analysis (PCA) was performed on the RNA-seq data normalized using DESeq2 package. The first two principal components (PC1 and PC2), which captured the largest amount of variance in the data, were selected for visualization and the PCA results were plotted using the ggplot2 package.^51^ To assess the relationship between the first few PCs (PC1 and PC2) and different variables (Cell type: Melanopsin vs. Empty and Stimulation: no stimulation, 1h and 12h), Kruskal-Wallis tests were used to determine if there were significant differences in PCs between the sub-groups. If significant differences were observed, post-hoc pairwise comparisons were performed using the Wilcoxon rank sum test. *p*-values from the pairwise tests were adjusted for multiple comparisons using the Benjamini-Hochberg method to control the false discovery rate.

##### Functional enrichment analysis

To elucidate the biological significance of the DEGs, we performed a comprehensive functional enrichment analysis using g:Profiler (version e111_e.g.,58_p18_f463989d, database updated on 25/01/2024).^45^ A list of DEGs with an adjusted *p*-value threshold of 0.05 was submitted to g:Profiler, to investigate Gene Ontology (GO) and Kyoto Encyclopedia of Genes and Genomes (KEGG) pathway-enriched terms. The analysis was performed using default parameters, except for the option to highlight only driver terms. Terms with an FDR of < 0.05 were considered statistically significant. Upstream transcription factors (TFs) of statistically significant DEGs (adjusted *p*-value < 0.05) were predicted using the ChIP-X Enrichment Analysis 3 (ChEA3) algorithm (https://maayanlab.cloud/chea3/).^33^ ChEA3 performs TF-target overrepresentation analysis by integrating data from six TF-target set libraries including: GTEx Coexpression, ReMap ChIP-seq, Enrichr Queries, ENCODE ChIP-seq, ARCHS4 Coexpression and Literature ChIP-seq. Predicted TFs were ranked based on their average rank across all TF-target gene libraries (MeanRank). The resulting TF-Gene regulatory networks were constructed and visualized using Cytoscape^50^ and made publicly available on the Network Data Exchange (NDEx) platform.^55^

##### Gene set enrichment analysis

To investigate the functional relevance of the transcriptomic data, Gene Set Enrichment Analysis (GSEA) was performed using the fgsea package in R (version 1.28.0).^46^ The input for GSEA was the ranked list of all genes from the DESeq2 differential expression analysis, ordered by the Wald test statistic. This approach evaluates gene set enrichment across the entire transcriptome, without applying a predefined threshold for differentially expressed genes.^56^ The analysis utilized the Hallmark gene sets (50 gene sets)^57^ from the Molecular Signatures Database (MSigDB), which were downloaded as a .gmt file from https://www.gsea-msigdb.org/gsea/msigdb/human/collections.jsp#H (accessed January 10, 2025). The fgsea package was executed with default parameters to calculate enrichment scores (ES), normalized enrichment scores (NES), and associated *p*-values. The adjusted *p*-values (FDR) were used to determine statistical significance, with gene sets having an FDR < 0.05 considered significantly enriched. Visualizations, including enrichment plots for significant pathways, were generated using the fgsea and ggplot2 packages in R.

##### Phosphoproteomics

**Sample preparation and protein digestion**: Cell pellets were re-suspended in 150 μL of lysis buffer (8 M Urea, 1% SDS, 50 mM Tris at pH 8.5 and protease and phosphatase inhibitor (Roche)). Cell lysis was done by vortexing and probe sonication on ice to destroy DNA (20% amplitude, 5/2 pulse in 40 s). Lysate was cleared by centrifugation at 4°C, 13k rpm for 10 minutes. Cleared lysate was transferred to a new tube. Proteins were reduced with DTT to a final concentration of 5 mM via incubation for 1 hour at 25°C and alkylated with iodoacetamide to a final concentration of 15 mM via incubation for 1 hour at 25°C in the dark. Excess iodoacetamide was quenched by adding 10 mM DTT. Methanol, chloroform, and water were added with volumes 4:1:3. Washing was done with one volume of methanol and a second wash with ice-cold methanol. Pellets were kept between washing steps and centrifugation at 15k rpm for 10 min. Pellets were air-dried (not completely dry). Protein pellets were dissolved in 8 M Urea and 50 mM Tris at pH 8.5 and diluted with equal volume of 50 mM Tris at pH 8.5 (final urea concentration 4 M). LysC was added (1:50; LysC:Protein) and incubated in a thermo-shaker, overnight with 300 rpm at 24°C. Next, three volumes of 50 mM Tris at pH 8.5 was added (final urea concentration 1 M). Trypsin (1:75; Trypsin:Protein) was added and incubated in a thermo-shaker, 300 rpm at 24°C for 6 h. Samples were centrifuged at 4°C, 15k rpm for 10 min; pellets were discarded and supernatants saved.

**Phosphopeptide enrichment**: Samples were desalted using C18 SepPak (Waters, 50 mg resin, 150 μL column volume, 5% binding capacity) and enriched for phosphopeptides using Pierce TiO2 Phosphopeptide Enrichment and Clean-up Kit (Thermo Scientific Pierce). Manufacturer's standard protocols were used.

**LC-MS/MS analysis**: Phosphopeptides were injected onto the nLC-MS/MS system, Ultimate 3000 RSLCnano chromatography system, and Q Exactive Plus Orbitrap mass spectrometer (Thermo Scientific). The peptides were separated on a homemade C18 column, 25 cm (Silica Tip 360μm OD, 75μm ID, New Objective, Woburn, MA, USA) with a 120 min gradient at a flow rate of 300 nL/min. The gradient went from 5 to 26% of buffer B (2% acetonitrile and 0.1% formic acid) in 115 min and up to 95% of buffer B in 5 min. The effluent was electro sprayed into the mass spectrometer directly via the column. A survey MS spectrum was acquired at a resolution of 140 000 in the range of m/z 300–1650. MS/MS data was obtained with a higher-energy collisional dissociation (HCD) at a resolution of 17 500.

**Peptide identification and quantification**: Raw files were processed and analyzed with Proteome Discoverer (Ver 3.0, Thermo Scientific). The data was matched against SwissProt Human database (20361 entries, May 2022) using Sequest as a search engine with a precursor tolerance of 5 ppm and a fragment ion tolerance of 0.02 Da. Tryptic peptides were accepted with 0 missed cleavage. Methionine oxidation and Serine, Threonine, Thyrosine phosphorylations were set as variable modifications, and cysteine carbamidomethylation was set as fixed modification. Percolator was used for PetideSpectraMatch validation with a strict FDR threshold of 1%. For label-free quantification, the Minora Feature Detector was used with trace length of 5 min, S/N of 1 and max delta RT of isotope pattern multiplets of 0.2 min. A Sequest HT threshold score of 2 was chosen for peptide validation. Only unique peptides were used for relative quantification, and proteins were required to pass a protein FDR of 1%.

**Missing value imputation and principal component analysis**: Based on a previous comparative study^58^ in label-free proteomics, reporting that random forest–based imputation consistently outperformed other popular methods, missing values were imputed using the missForest R package^48^ with 100 trees. To evaluate the overall variability among samples, PCA was performed on the normalized protein and phosphopeptide count data. The first two/three principal components (PC1, PC2 and/or PC3), accounting for the largest proportion of variance, were selected for visualization, and the PCA results were plotted using the ggplot2 package.

**Differential abundance analysis**: Differential abundance analysis of both proteomics and phosphoproteomics datasets was performed using the limma package in R.^49^ A design matrix was generated from the imputed, normalized protein and phosphopeptide count data, along with the associated sample metadata, using the model formula (∼ Cell type + Stimulation) to account for the effects of both cell type and stimulation. In this context, “stimulation” refers to one hour of melanopsin-activating light pulses delivered either once per minute (15 mHz) or once every two minutes (8 mHz). Statistical testing was then conducted, and significantly differentially abundant proteins (DAPs) and differentially abundant phosphopeptides (DAPPs) were identified based on an FDR threshold of < 0.05.

**Functional enrichment analysis**: To explore the biological relevance of the DAPs, functional enrichment analysis was conducted using g:Profiler (version e111_e.g.,58_p18_f463989d; database updated on 25 January 2024). DAPs with an adjusted *p*-value < 0.05 were submitted to g:Profiler to identify enriched GO terms and KEGG pathways. The analysis was performed with default parameters, except that only driver terms were highlighted. Enrichment results with an FDR < 0.05 were considered statistically significant.

**Kinase enrichment analysis**: Upstream kinases associated with statistically significant differentially abundant phosphopeptides (adjusted *p*-value < 0.05) were predicted using the Kinase Enrichment Analysis 3 (KEA3) tool (https://maayanlab.cloud/kea3/).^47^ KEA3 identifies kinases whose known or predicted substrates are overrepresented in the input list by integrating multiple kinase–substrate interaction libraries, including experimentally validated and computationally inferred datasets. Predicted kinases were ranked based on their average position (MeanRank) across all available libraries. The HUGO Gene Nomenclature Committee (HGNC)-approved gene symbols corresponding to each phosphopeptide’s protein identifier were retrieved using the biomaRt^59^ R package and used as input for KEA3.

**Network construction**: The top ten enriched TFs at 1h stimulation, predicted by ChEA3 (based on MeanRank; listed in Table 1, ChEA3 section), were used as input to retrieve their interactors. “Transcription factor interactors” refer to any molecules reported to physically interact with the given TF in the BioGRID^53^ protein–protein interaction database (version 4.4.248, released August 2025; https://thebiogrid.org). In BioGRID, the search was restricted to *Homo sapiens* and filtered to include only interactions supported by low-throughput (LTP) physical evidence, retaining higher-confidence interactions. Relevant transcription factor interactors are defined here as those interactors shared among at least two of the top transcription factors. Upstream kinases associated with these interactors were then predicted using the KEA3 tool. The resulting Expression-Kinases regulatory network was constructed and visualized using Cytoscape^50^ and made publicly available on the NDEx platform.^55^

### Quantification and statistical analysis

All experiments were performed using independent biological replicates unless otherwise stated. Statistical details for individual experiments, including exact *n* values, statistical tests, and significance levels, are provided in the corresponding figure legends and Results sections. N denotes sample size with independent cultures (biological replicates). For luciferase assays, firefly luciferase values were normalized to Renilla luciferase activity and expressed relative to the indicated unstimulated control condition. RT-qPCR data were normalized to GAPDH using the ΔΔ CT-method. Statistical analyses were performed in MATLAB (MathWorks) or R (R Foundation for Statistical Computing). Comparisons involving multiple experimental groups were analyzed using ANOVA as appropriate, followed by Tukey’s post hoc multiple-comparison test. For two-group comparisons, unpaired or paired two-tailed Student’s t tests were used where applicable. For RNA sequencing and proteomics/phosphoproteomics analyses, statistical testing and multiple-testing correction were performed using DESeq2 and limma as described above, with false discovery rate (FDR)-adjusted *p* values < 0.05 considered statistically significant. Data are presented as mean ± SEM, where the mean represents the measure of central tendency and SEM represents the measure of dispersion, unless otherwise stated. For all statistical analyses, *p* < 0.05 was considered statistically significant unless otherwise specified. No statistical methods were used to predetermine sample size. Samples and experimental groups were not randomized, and no stratification procedures were applied unless otherwise stated. No data points or samples were excluded from the analyses unless predefined technical quality-control criteria were not met. For proteomic and phosphoproteomic analyses, one biological replicate from the 15 mHz stimulation condition did not pass quality-control criteria and was excluded from the final analysis. Consequently, sample sizes for these datasets ranged from n = 2–3 biological replicates per condition. Statistical tests were selected based on the experimental design and data structure. Assumptions of the applied statistical tests were considered based on the experimental design and distribution of the data.

## Supplemental information

Document S1. Figures S1–S6

Table S1. Differentially expressed genes identified between fast and slow stimulation regimes at 1 h, related to Figure 4

Table S2. Differentially expressed genes identified between fast and slow stimulation regimes at 12 h, related to Figure S5

Table S3. Genes exhibiting significant cell-type-dependent differences between fast and slow stimulation regimes identified by interaction-model analysis after 1 h of stimulation

Table S4. Differentially abundant proteins identified between fast (15 mHz) and slow (8 mHz) melanopsin stimulation regimes after 1 h of stimulation, related to Figure 6

Table S5. Differentially abundant phosphopeptides identified between fast (15 mHz) and slow (8 mHz) melanopsin stimulation regimes after 1 h of stimulation, related to Figure 6
