# Supplementary material for: Temporal patterns of melanopsin-evoked calcium signaling engage NF-κB-associated transcriptional and phosphoproteomic responses
Source: iScience. 2026 Oct 26;29(11):118031. doi: 10.1016/j.isci.2026.118031 (PMC13734797; doi:10.1016/j.isci.2026.118031)
Supplement: Supplementary file 1 [file mmc1.pdf]

iScience, Volume *29*

Supplemental information

Temporal patterns of melanopsin-evoked calcium signaling engage NF-κB-associated transcriptional and phosphoproteomic responses

Parvaneh Nikpour, Manuel Varas-Godoy, Johan Tenghamn, Per Uhlén, and Erik Smedler

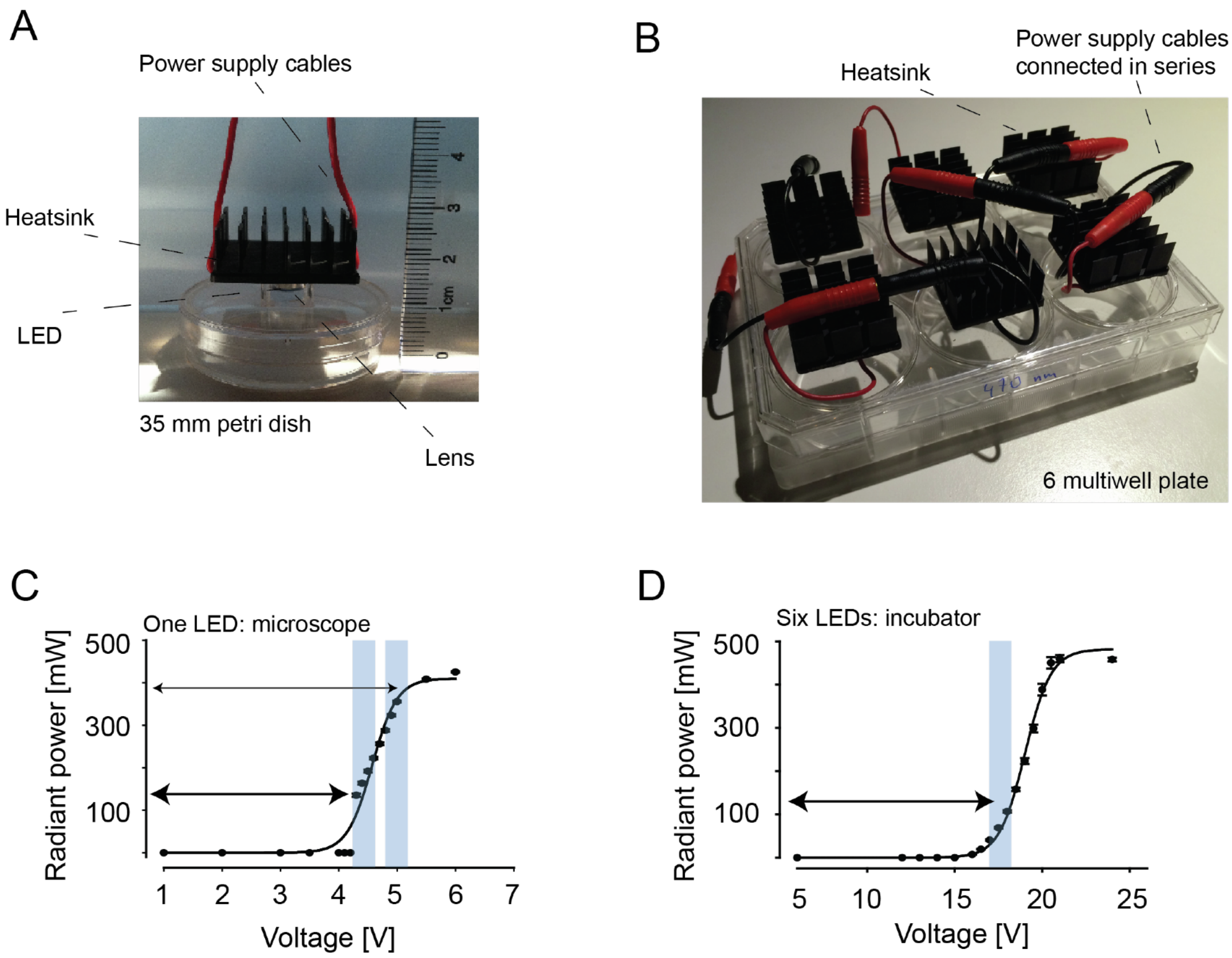

Figure S1. Characterization of the LED-based light stimulation setup

A) Picture of LED with cables used for stimulation under microscope and B) in incubator. Light power as a function of driver voltage for C) one LED or D) six LEDs connected in series. Average and SEM for N=3.

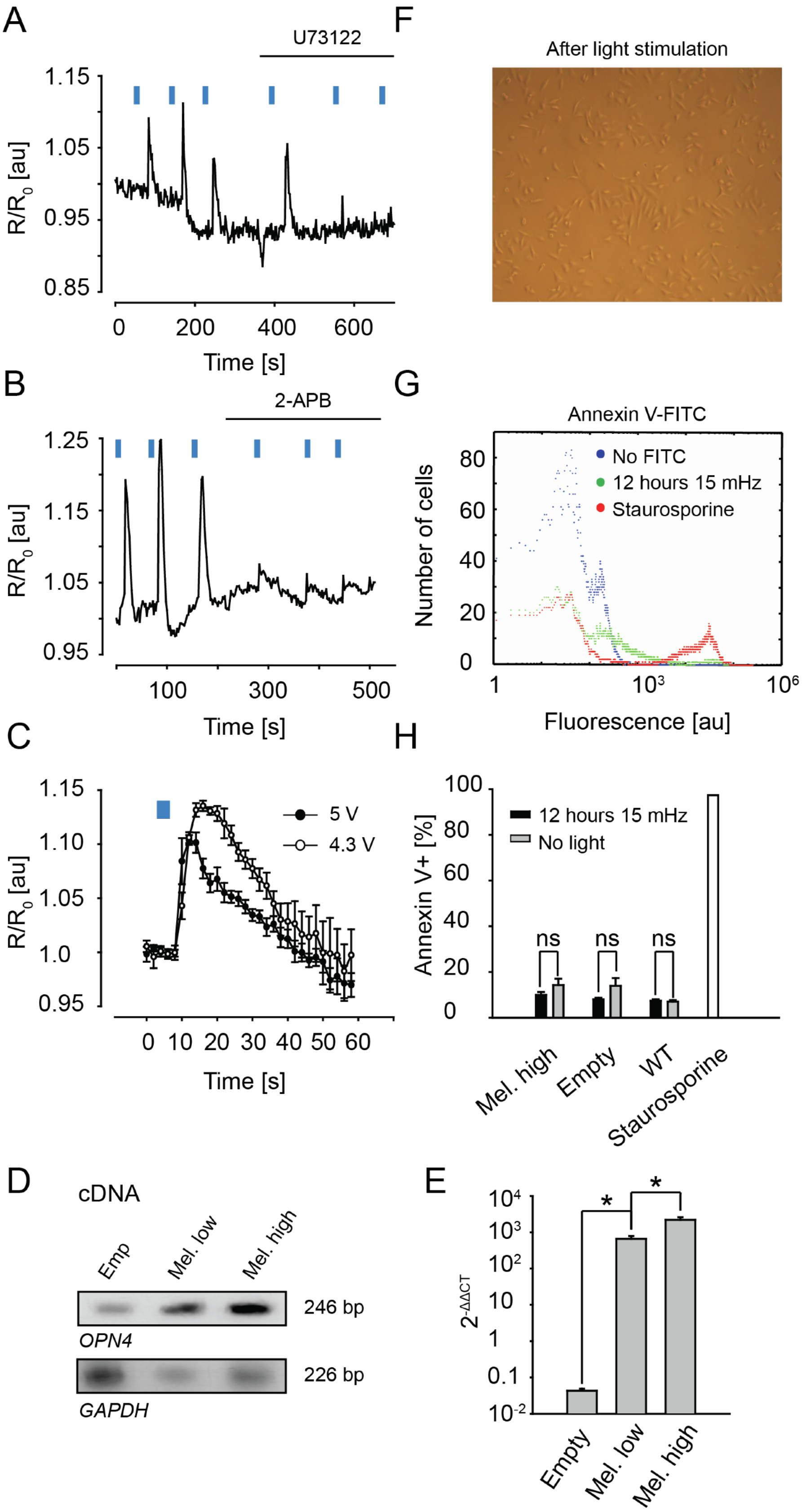
A
U73122
1.15
1.05
0.95
0.85
R/R0 [au]
0
200
400
600
Time [s]
B
2-APB
1.25
1.15
1.05
0.95
R/R0 [au]
100
300
500
Time [s]
C
1.15
1.10
1.05
1.0
0.95
R/R0 [au]
5 V
4.3 V
0 10 20 30 40 50 60
Time [s]
D
cDNA
Emp
Mel. low
Mel. high
246 bp
OPN4
226 bp
GAPDH
E
10^4
10^3
10^2
10
1
0.1
10^-2
2^-ΔΔCT
*
*
Empty
Mel. low
Mel. high
F
After light stimulation
G
Annexin V-FITC
80
60
40
20
0
Number of cells
No FITC
12 hours 15 mHz
Staurosporine
1
10^3
10^6
Fluorescence [au]
H
100
80
60
40
20
0
Annexin V+ [%]
12 hours 15 mHz
No light
ns
ns
ns
Mel. high
Empty
WT
Staurosporine

Figure S2. Validation of melanopsin-evoked calcium signaling and stable melanopsin-expressing cells

A) Ca^{2+} response to light stimulation is abolished by PLC inhibition with 5 µM of U73122 or B) 100 µM of IP3R inhibition by 2-APB. C) Ca^{2+} response to light stimulation with either low (4.3 V) or high (5 V) driving voltage. Average and SEM for four cells D-E) Strong fluorescence corresponds to strong expression of melanopsin as assayed by qPCR. The resulting amplicon sizes were 226 bp for *GAPDH* and approximately 246 bp for *OPN4* (melanopsin). F) Normal qualitative cell morphology after twelve hours of light stimulation. G-H) No increase in early apoptotic cells as assayed by Annexin V staining after light stimulation. P values were calculated with unpaired student's t test, average and SEM for N=3. * $p<0.05$.

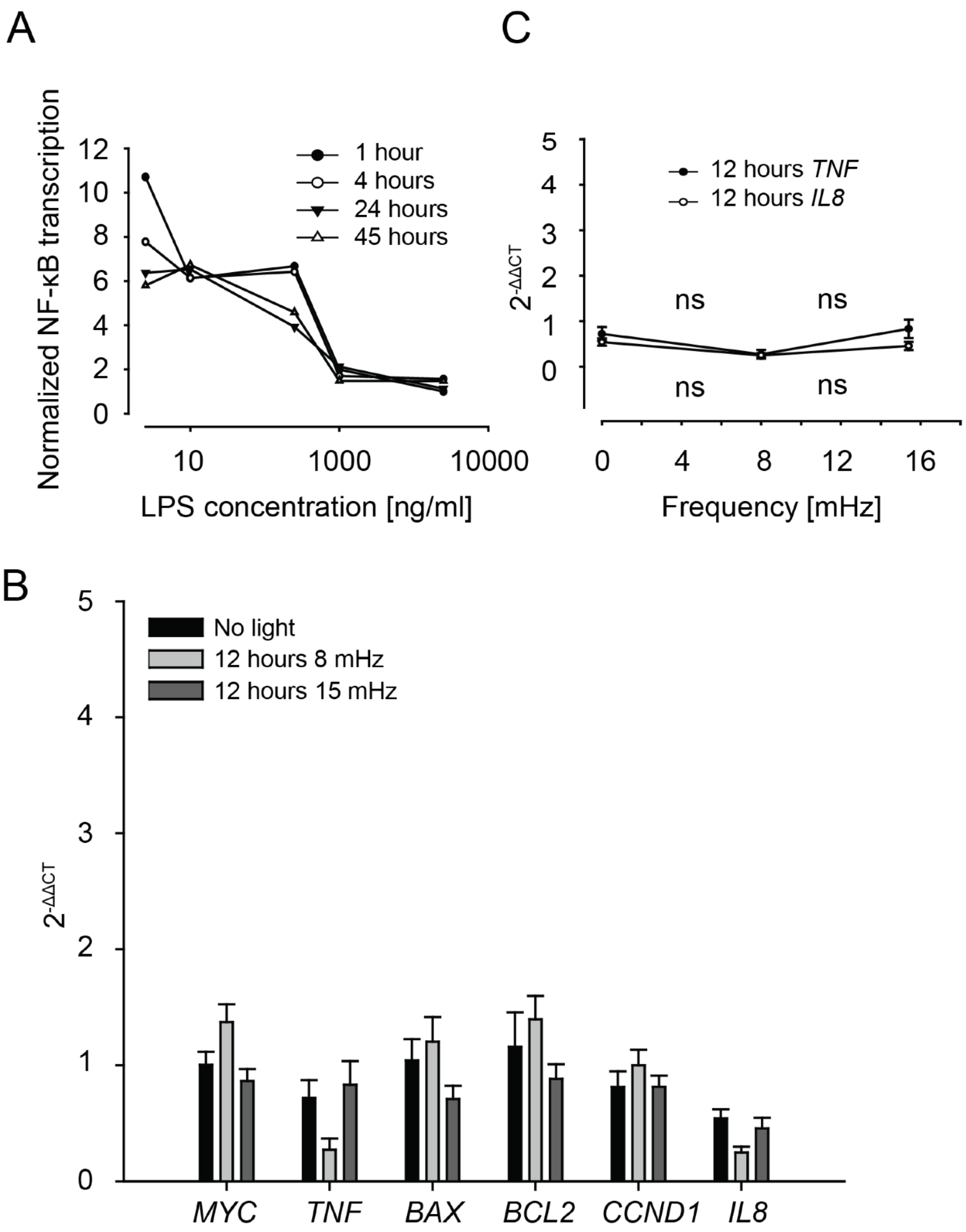

Figure S3. NF-κB-associated responses to LPS and prolonged melanopsin stimulation

A) NF-κB-associated transcription as measured by luciferase assay following LPS stimulation at concentrations ranging from 0.05–10 μg/mL and varying exposure durations. Firefly luciferase activity was normalized to Renilla luciferase and expressed relative to unstimulated controls. These experiments were included as positive-control validation of NF-κB reporter responsiveness. B) Gene expression of a set of genes dependent on NFκB as measured by RT-qPCR after 12 hours of light stimulation. C) Expression of *TNF* and *IL8* as function of frequency. P values were calculated with unpaired student's t test, average and SEM for N=3. * $p<0.05$.

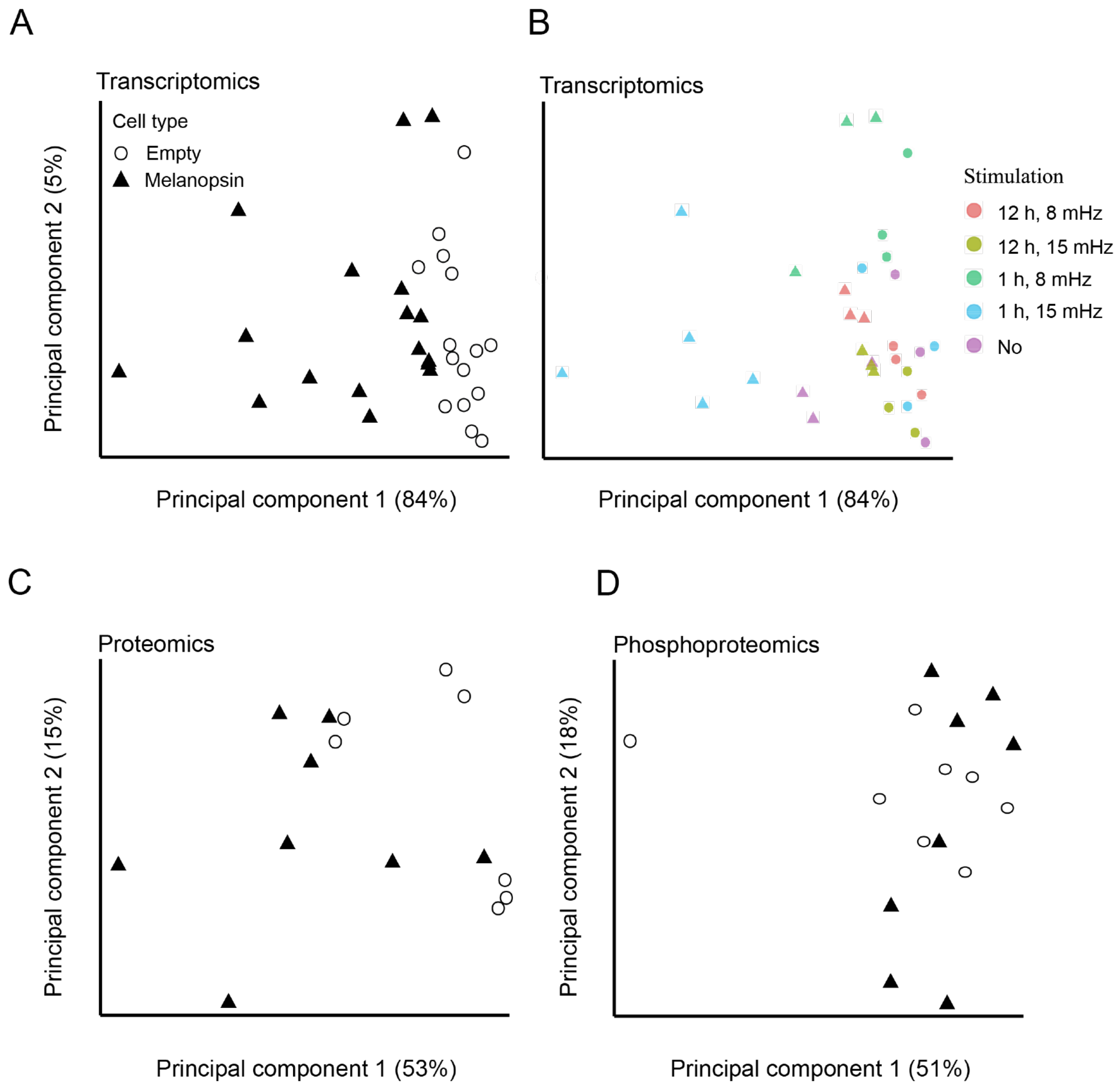

Figure S4. Principal component analysis of transcriptomic, proteomic, and phosphoproteomic datasets

A) Principal Component Analysis (PCA) of RNA-seq samples based on normalized gene expression counts. The PCA plot displays the distribution of 32 RNA-seq samples across the first two principal components, which explain 84% and 5% of the total variance, respectively. Samples are categorized by Cell_type, with circles (○) representing Empty controls and triangles (▲) representing Melanopsin-expressing cells. B) Additionally, samples are grouped by their Stimulation conditions, with the following color scheme: Red for 12 h if 8 mHz, Yellow for 12 h of 15 mHz, Purple for 1 h of 8 mHz, Green for 1 h of 15 mHz and Cyan for No stimulation. (C) PCA of proteomics data projected onto PC1 (52.59% variance) and PC2 (14.91% variance) shows limited separation between groups. (D) PCA of phosphoproteomics data projected onto PC1 (51.19% variance) and PC2 (17.89% variance) shows no clear group separation.

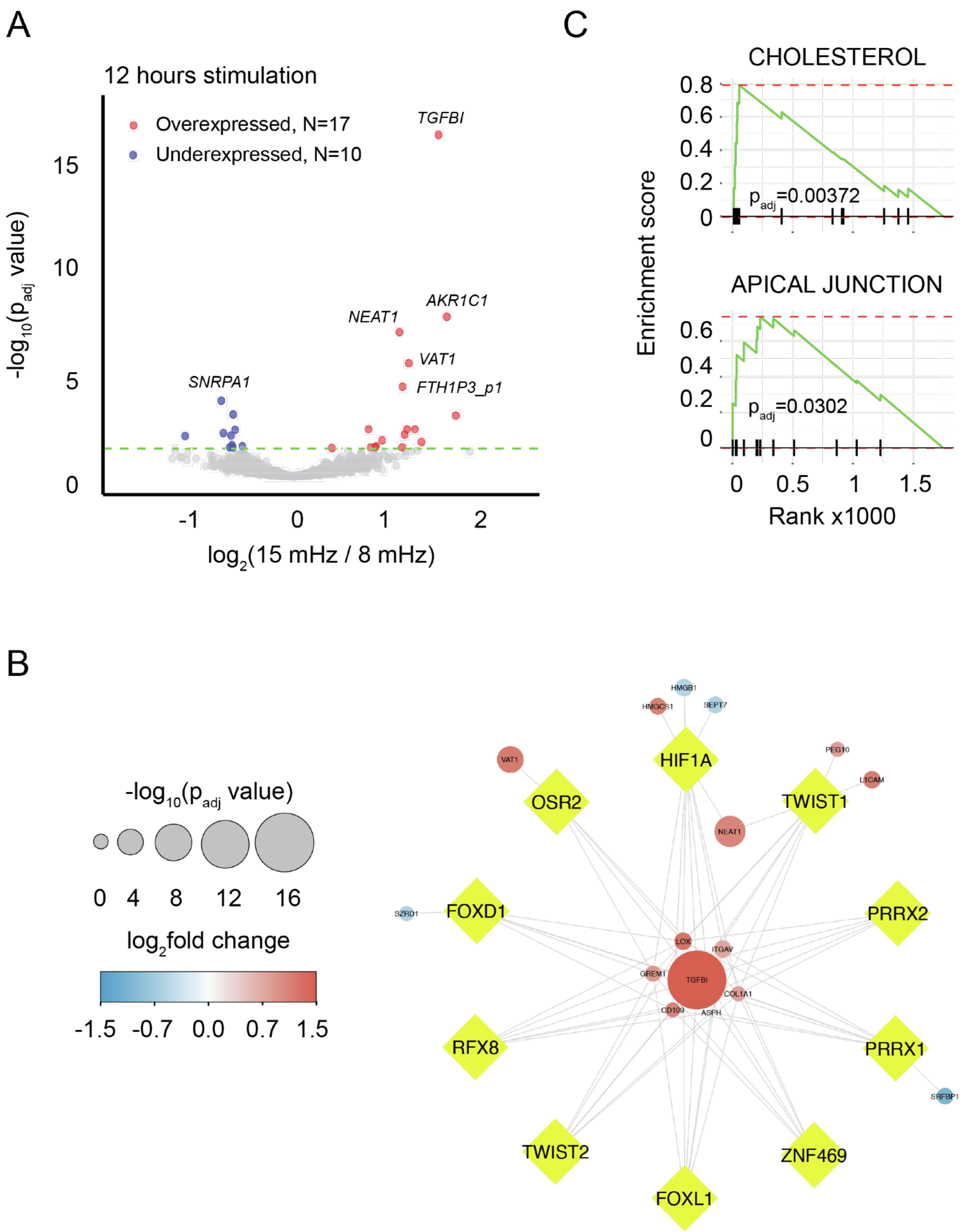

Figure S5. Transcriptomic responses to fast and slow melanopsin stimulation after 12 hours

A) Volcano plot of bulk RNAseq data for 12 hours of stimulation showing the $\log_2$ fold change (x-axis) against the $-\log_{10}$ adjusted p-value (y-axis). Genes with adjusted p-value < 0.05 are highlighted: over-expressed genes are marked in red, and under-expressed genes are marked in blue. Non-significant genes are shown in grey. N = 3 for independent cultures. B) TF-gene regulatory network constructed using (DEGs) after 12 hours of stimulation. Regulatory interactions are computationally inferred using ChEA3. TFs are represented as yellow diamond-shaped nodes, while target genes are shown as circles. Node size corresponds to $-\log_{10}$ adjusted p-value, with color representing $\log_2$ fold change ($\log_2$FC): red

for upregulated genes and blue for downregulated genes. Edges indicate regulatory interactions between TFs and their target genes. C) Enrichment plots of the gene set enrichment analysis results of transcriptomic data. Enrichment plots display the significant pathways identified by GSEA for 12 h using the Hallmark gene sets from MSigDB. The x-axis represents the rank order of genes in the list, while the y-axis indicates the running enrichment score. Tick marks along the x-axis denote the positions of genes from the respective gene set in the ranked list. Pathways with FDR < 0.05 were considered significantly enriched.

A

1 hour stimulation

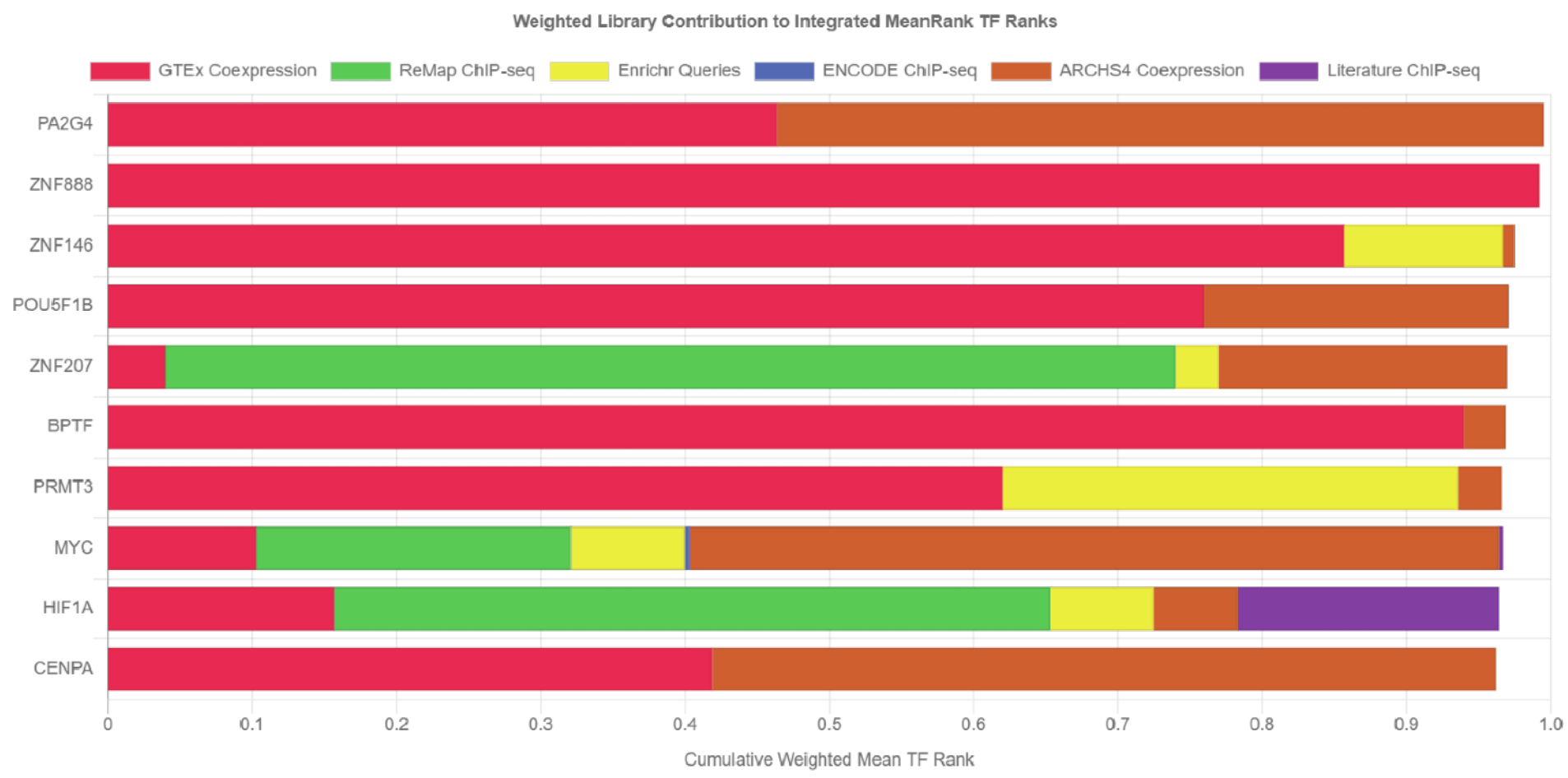

B

12 hours stimulation

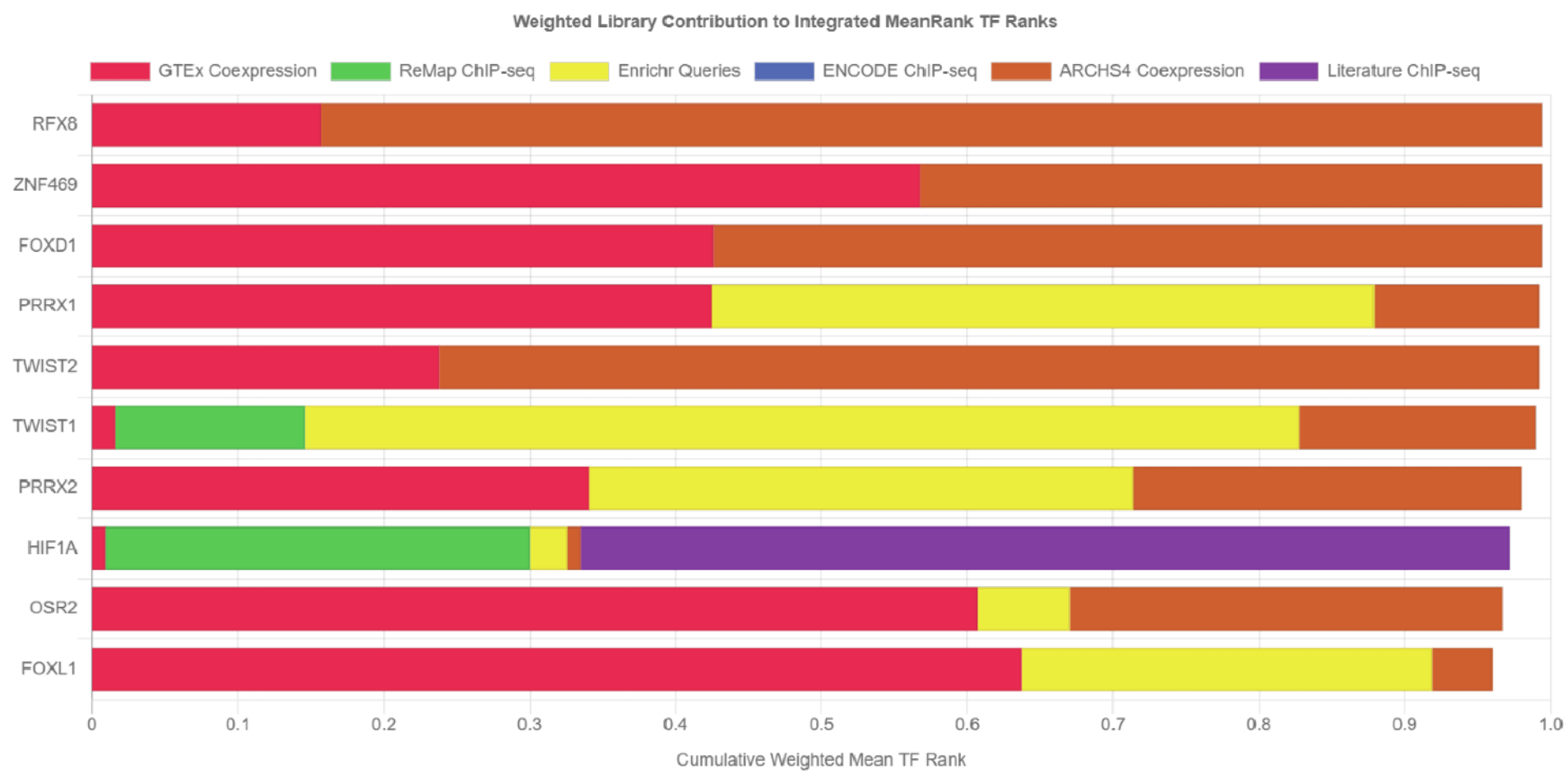

Figure S6. Transcription factor enrichment following fast versus slow melanopsin stimulation

Bar charts showing the cumulative weighted mean rank of the top 10 transcription factors (TFs) identified by ChEA3 analysis of DEGs after 1 (A) and 12 (B) hours of stimulation under fast (15 mHz) versus slow (8 mHz) melanopsin stimulation conditions. The x-axis represents the cumulative weighted mean TF rank, while the y-axis lists the top-ranked TFs. The colored segments indicate the contribution of different evidence sources to each TF ranking: GTEx Coexpression (red), ReMap ChIP-seq (green), Enrichr Queries (yellow), ENCODE ChIP-seq (blue), ARCHS4 Coexpression (orange), and Literature ChIP-seq (purple).
